# *In Vitro* Modeling of the Bipolar Disorder and Schizophrenia Using Patient-Derived Induced Pluripotent Stem Cells with Copy Number Variations of *PCDH1*5 and *RELN*

**DOI:** 10.1523/ENEURO.0403-18.2019

**Published:** 2019-10-16

**Authors:** Takaya Ishii, Mitsuru Ishikawa, Koki Fujimori, Takuji Maeda, Itaru Kushima, Yuko Arioka, Daisuke Mori, Yuhki Nakatake, Bun Yamagata, Shintaro Nio, Takahiro A. Kato, Nan Yang, Marius Wernig, Shigenobu Kanba, Masaru Mimura, Norio Ozaki, Hideyuki Okano

**Affiliations:** 1 Department of Physiology, Keio University School of Medicine, Shinjuku-ku, Tokyo 160-8582, Japan; 2 iPS Cell-Based Drug Discovery, Drug Research Division, Sumitomo Dainippon Pharma. Co., Ltd, Osaka, Osaka 554-0022, Japan; 3 Department of Psychiatry, Nagoya University Graduate School of Medicine, Aichi, Nagoya 466-8550, Japan; 4 Institute for Advanced Research, Nagoya University, Aichi, Nagoya 466-8550, Japan; 5 Medical Genomics Center, Nagoya University Hospital, Aichi, Nagoya 466-8550, Japan; 6 Department of Systems Medicine, Keio University School of Medicine, Shinjuku-ku, Tokyo 160-8582, Japan; 7 Department of Neuropsychiatry, Keio University School of Medicine, Shinjuku-ku, Tokyo 160-8582, Japan; 8 Department of Neuropsychiatry, Graduate School of Medical Sciences, Kyushu University, Fukuoka, Fukuoka 812-8582, Japan; 9 Institute for Stem Cell Biology and Regenerative Medicine and Department of Pathology, Stanford University School of Medicine, Stanford, California 94305; 10 Department of Neuroscience, Friedman Brian Institute, Black Family Stem Cell Institute, Icahn School of Medicine at Mount Sinai, New York 10029

**Keywords:** bipolar disorder, copy number variations, GABAergic neurons, glutamatergic neurons, induced pluripotent stem cells, schizophrenia

## Abstract

Bipolar disorder (BP) and schizophrenia (SCZ) are major psychiatric disorders, but the molecular mechanisms underlying the complicated pathologies of these disorders remain unclear. It is difficult to establish adequate *in vitro* models for pathological analysis because of the heterogeneity of these disorders. In the present study, to recapitulate the pathologies of these disorders *in vitro*, we established *in vitro* models by differentiating mature neurons from human induced pluripotent stem cells (hiPSCs) derived from BP and SCZ patient with contributive copy number variations, as follows: two BP patients with *PCDH15* deletion and one SCZ patient with *RELN* deletion. Glutamatergic neurons and GABAergic neurons were induced from hiPSCs under optimized conditions. Both types of induced neurons from both hiPSCs exhibited similar phenotypes of MAP2 (microtubule-associated protein 2)-positive dendrite shortening and decreasing synapse numbers. Additionally, we analyzed isogenic *PCDH15*- or *RELN*-deleted cells. The dendrite and synapse phenotypes of isogenic neurons were partially similar to those of patient-derived neurons. These results suggest that the observed phenotypes are general phenotypes of psychiatric disorders, and our *in vitro* models using hiPSC-based technology may be suitable for analysis of the pathologies of psychiatric disorders.

## Visual Abstract

**Figure:**
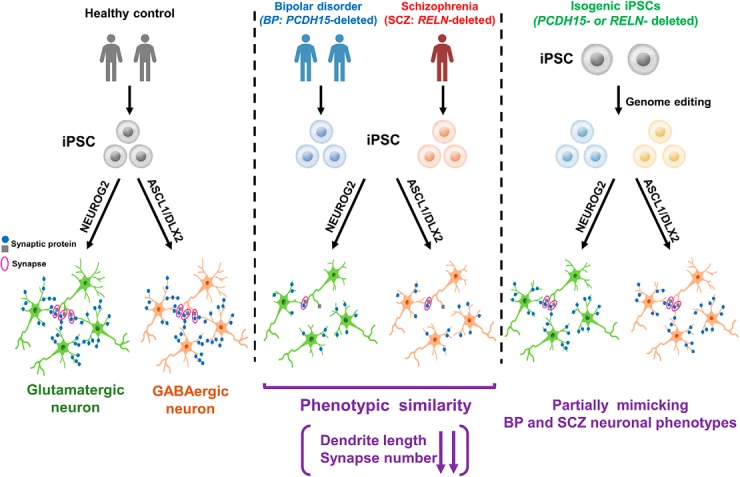

## Significance Statement

Useful *in vitro* models of psychiatric disorders such as schizophrenia and bipolar disorder are urgently required for pathological analysis and drug discovery. In this study, mature excitatory and inhibitory neurons were induced from patient-derived induced pluripotent stem cells. The patient-derived induced neurons exhibited abnormalities in dendrite and synapse formation *in vitro*, which are similar to the previously reported findings observed in postmortem brains. Our *in vitro* model may reflect general phenotypes of psychiatric disorders and can be used to further examine therapeutic targets.

## Introduction

Both bipolar disorder (BP) and schizophrenia (SCZ) are chronic and debilitating psychiatric disorders that affect ∼1% of the worldwide population (McGrath et al., 2008; Grande et al., 2016). Although these disorders are highly heritable (Craddock and Sklar, 2013; Millan et al., 2016), the molecular mechanisms underlying the complex pathology of these disorders remain to be elucidated.

There are limitations to the recapitulation of clinical characteristics in animal models and postmortem brain studies because of genetic heterogeneity (O'Shea and McInnis, 2016; Prytkova and Brennand, 2017). Therefore, reliable models that functionally mimic live human brains are sought after. Induced pluripotent stem cells (iPSCs) are expected to become a promising tool for recapitulating disease-specific phenotypes *in vitro* (Okano and Yamanaka, 2014; O'Shea and McInnis, 2016; Watmuff et al., 2016; Prytkova and Brennand, 2017; Tobe et al., 2017). Although recent studies established iPSCs from BP and SCZ patients and induced neurons to analyze phenotypes (O'Shea and McInnis, 2016; Prytkova and Brennand, 2017; Wen, 2017), the maturity and subtype specificity of induced neurons remain to be considered. Thus, analysis of mature and subtype-specific neurons is required for further elucidation of the pathologies. It has been suggested that the collapse of the excitation–inhibition (E/I) balance plays key roles in BP and SCZ (Gao and Penzes, 2015; Lee et al., 2018). Therefore, it is important to focus on certain neurons that are the main players in the E/I balance, such as glutamatergic neurons and GABAergic neurons. Recent studies have shown that transcription factor overexpression enabled iPSCs to be differentiated into specific neurons, including glutamatergic neurons (Zhang et al., 2013) and GABAergic neurons (Colasante et al., 2015; Yang et al., 2017).

Many genetic mutations are associated with these disorders, especially copy number variations (CNVs), which are important contributive factors that affect the onset and treatment resistance of BP and SCZ (Georgieva et al., 2014; Green et al., 2016; Kushima et al., 2017). Thus, to analyze the pathologies, we used iPSC lines derived from patients who carried certain CNVs: two BP patients with *PCDH15* exonic deletions and an SCZ patient who carried an *RELN* exonic deletion. Protocadherin 15 (PCDH15), encoded by *PCDH15,* is a member of the cadherin superfamily. *PCDH15* mutations cause Usher syndrome, which results in hearing vision loss (Ahmed et al., 2001; Alagramam et al., 2001; Kim et al., 2011). A recent genome-wide association study suggested that *PCDH15* is associated with psychiatric disorders (Lo et al., 2017). In addition, *de novo* or rare exonic deletions in *PCDH15* were identified in BP patients (Georgieva et al., 2014; Noor et al., 2014). These studies suggested that *PCDH15* is a risk gene for psychiatric disorders. Reelin, which is encoded by *RELN*, is an extracellular matrix protein that regulates brain developmental processes, such as neuronal migration and dendrite formation, and modulates synaptic functions in adult brains (Ishii et al., 2016; Lee and D'Arcangelo, 2016). Reelin is associated with various psychiatric disorders such as SCZ, autism spectrum disorders, and BP (Folsom and Fatemi, 2013; Ishii et al., 2016). Two SCZ patients with exonic deletions in *RELN* have been reported in previous studies (Costain et al., 2013; Kushima et al., 2017).

In this study, to recapitulate the pathologies in BP and SCZ *in vitro*, we differentiated patient-derived iPSCs into neurons and identified a set of similar phenotypes among induced neurons derived from patients with different genetic lesions, which potentially represents a cell biological basis for shared clinical features between these two different disorders.

## Materials and Methods

### Subjects

A human subject was recruited at Keio University, and another from Kyushu University. Written informed consent for the present study was obtained from this patient. All the experimental procedures for biopsy, genetic analysis, and iPSC production were approved by the Keio University School of Medicine Ethics Committee (approval #20080016), the Ethics Committee of Nagoya University (approval #2010-1033-3 and #2012-0184-14), and the Ethics Committee of Kyushu University (approval #IRB-30-537).

We recruited two patients with BP. The first patient (BP1) was a 43-year-old Japanese female with a family history of alcohol use disorder. Her developmental history was unremarkable. In her early thirties, she had depressive moods and suicidal thoughts. Although she was treated with antidepressant medications, a significant treatment effect was not observed. After 3 years, she received a diagnosis of bipolar I disorder because she had her first manic episode, which included an elevated mood, increased activity and talkativeness, and decreased need for sleep. In her late thirties, she became depressed again after she received a diagnosis of a malignant tumor. Her depressive symptoms were chronic and refractory to pharmacological treatments, requiring multiple hospitalizations. At the time of the study evaluation, she was receiving treatment with lithium and quetiapine.

The second patient (BP2) was a 57-year-old Japanese female with no remarkable developmental history and no family history of psychiatric disorders. She had been committed to various social activities and was sometimes hyperactive until her middle thirties (hypomanic episodes). Since her late thirties, she had experienced insomnia, depressive moods, and severe suicidal thoughts two or three times a year, requiring multiple hospitalizations. Despite treatment with antidepressant medications, a considerable treatment effect was not observed. After she had received a diagnosis of bipolar I disorder based on her life history and hyperactivity between depressive episodes, lithium was prescribed. However, the response was still poor. At age 50 years, lithium was completely replaced with valproate and lamotrigine. After that, her mood stabilized and no subsequent hospitalizations were necessary. At the time of the study evaluation, she was still receiving valproate and lamotrigine. Although she had once received a diagnosis of bipolar I disorder, the diagnosis was revised to bipolar II disorder because of the lack of severe manic episodes.

### iPSC generation and culture

The 1210B2 (Nakagawa et al., 2014), 201B7 (Takahashi et al., 2007), WD39 (Imaizumi et al., 2012), 414C2 (Okita et al., 2011), eKA3 (Matsumoto et al., 2016), eTKA4 (Matsumoto et al., 2016), and NC1032-1-2 cell lines were used as the healthy control human iPSC lines. The NC1032-1-2 line was established from cells obtained from a healthy 30-year-old Japanese woman using episomal vectors as previously described (Okita et al., 2013), which was then used for establishment of the isogenic PCDH15-deleted iPSC line. iPSC lines derived from an SCZ patient with heterozygous *RELN* deletion (SCZ1-1, SCZ1-2) were established in a previous study (Arioka et al., 2018). BP patient-derived iPSCs (BP-iPSCs) were generated by a previously reported method (Okita et al., 2013; Hosoya et al., 2017). Briefly, episomal plasmids encoding six factors (*OCT4*, *SOX2*, *KLF4*, *LIN28*, *L-MYC*, *EBNA1*, and dominant-negative *p53*) were transduced into human peripheral blood mononuclear cells obtained from the patient. Established iPSCs were evaluated based on the expression of pluripotent markers, episomal transgene elimination, and the absence of clinically significant CNVs.

iPSCs were cultured with or without feeder cells. For on-feeder culture, iPSCs were grown on mitomycin-C-treated SNL murine fibroblast feeder cells in standard human pluripotent stem cell medium (DMEM/F12 medium, FUJIFILM Wako Pure Chemical) containing 20% KnockOut Serum Replacement (KSR; Thermo Fisher Scientific), 0.1 mm nonessential amino acids (NEAAs; Merck), 0.1 mm 2-mercaptoethanol (Merck), and 4 ng/ml fibroblast growth factor 2 (PeproTech) at 37°C in an atmosphere containing 3% CO_2_. For the feeder-free culture, iPSCs were grown on tissue culture dishes coated with 1 μg/ml iMatrix-511 (Laminin-511 E8, Nippi). iPSCs were cultured in StemFit AK02N (Ajinomoto) at 37°C in an atmosphere containing 5% CO_2_.

### Identification and validation of an exonic deletion of *PCDH15*

Genomic DNA was extracted from blood or iPSC lines. To identify an exonic deletion of *PCDH15*, we performed array comparative genomic hybridization (aCGH) using an SurePrint G3 Human CGH Microarray, 2 × 400K (Agilent). The experiment was performed following the manufacturer instructions. CNV calls were made with Nexus Copy Number software version 9.0 (BioDiscovery) using the Fast Adaptive States Segmentation Technique 2 algorithm, which is a hidden Markov model-based approach. The log2 ratio thresholds for copy number loss and copy number gain were set at −0.7 and 0.45, respectively. The significance threshold *p* value was set at 1 × 10^−6^, and at least four contiguous probes were required for CNV calls. To validate the exonic deletion of *PCDH15*, quantitative real-time PCR was performed with TaqMan copy number assay (Hs03733966_cn for BP1 and Hs00117569_cn for BP2; Applied Biosystems). All genomic locations are given in NCBI36/hg18.

### *In vitro* three-germ differentiation via embryoid body formation

To check the pluripotency of iPSCs, iPSCs treated with TrypLE Select (Thermo Fisher Scientific) were dissociated into single cells and plated in low-cell adhesion 96-well plates with V-bottomed conical wells. The cells were cultured in embryoid body (EB) medium (DMEM/F-12 containing 5% KSR, 2 mm l-glutamine, 1% NEAAs, and 0.1 mm 2-ME). On day 7, EBs were plated on culture plates coated with 0.1% gelatin (Merck) and 10 μg/ml fibronectin (Merck). The plated EBs were cultured for 3 weeks at 37°C in an atmosphere containing 5% CO_2_.

### qRT-PCR

RNA was extracted from the cells using the RNeasy Kit (Qiagen). One hundred nanograms of RNA was used to prepare cDNA using the iScript cDNA Synthesis Kit (Bio-Rad). qRT-PCR was performed with SYBR Premix Ex TaqII (TaKaRa Bio) on the ViiA 7 real-time PCR system (Thermo Fisher Scientific). Values were normalized to *GAPDH* levels. The comparative (ΔΔCt) method was used to analyze the data. Relative expression levels are presented as geometric means ± geometric SDs. The primers used for qRT-PCR were as follows: *GAPDH*: forward, AATCCCATCACCATCTTCCA; reverse, TGGACTCCACGACGTACTCA; *PCDH15* (set1): forward, GAGGCAGCCTTGGCAAGAAA; reverse, CTGTCGAAACATCTTCTGTCAAAGT; *PCDH15* (set2): forward, GGGACCATGGTTGGTGTAAT; reverse, CACCTGTGATGTTATTAATTCCAAA; and *RELN*: forward, AGAAGGACAAGACTCACAATGCT; reverse, GCTTCACAACCCACCACAAT.

### Neuron differentiation via dual SMAD inhibitor-treated EB formation

For dual SMAD inhibitor-treated EB (DSi-EB) formation, the cells were cultured in medium hormone mix (MHM; Shimazaki et al., 2001; Okada et al., 2004) containing B27 supplement (Thermo Fisher Scientific) with 5 μm SB431542 (Tocris Bioscience), 150 nm LDN193189 (StemRD), and 2.5 μm IWP-2 (Merck; Imaizumi et al., 2015). On day 7, DSi-EBs were plated on culture plates coated with Matrigel (Corning). The medium was replaced every 3 d.

### Plasmids construction

The *NEUROG2* expression vector (PB-TET-PH-lox66FRT-NEUROG2; Matsushita et al., 2017) was a gift from Dr. Minoru S.H. Ko (Keio Uniersity,Tokyo, Japan). *ASCL1* and *DLX2* expression vectors [PB-P(tetO)-h*Ascl1*-pA-PGK-PuroTK-pA and PB-P(tetO)-hDLX2-pA-floxPGKneo-pA] were established by Gateway cloning (Thermo Fisher Scientific). First, three plasmids with att-cloning sites (pProF-PHtetO_2_, pMK-pA-PGKpacTK-pA1, pMK-pA-floxPGKneo-pA) were constructed according to the manufacturer protocol for the MultiSite Gateway Three-Fragment Vector Construction Kit (Thermo Fisher Scientific) from pDONR-P4P1r and pDONR-P2rP3. Primer sequences and templates are listed below, as follows:

pProF-PHtetO_2_ (template: PB-TET-PH; Matsushita et al., 2017), primers: ggggACAACTTTGTATagaaaaGTTGttaattaagtcgacATTAAGTTGGGTAACGCCAGGG, ggggACTGCTTTTTTGTACAAACTTgcgatcgcGATGGCCGCCACCGCGGAGGC); pMK-pA-PGKpacTK-pA1 (template: pUC19PGKpacDeltaTKpA), primers: ggggACAGCTTTCTTGTACAAAGTGGTTAATTAAGGATCGGCAATAAAAAGACAGAATAAAACGCACGGGTGTTGGGTCGT, gggACAACTTTGTATAATAAAGTTGCCCGGGTGCATGCCTGCAGGTCGACTCTAGA);pMK-pA-floxPGKneo-pA (template: pL452), primers: ggggACAGCTTTCTTGtacaaaGTGGggatcggcaataaaaagacagaataaaacgcacgggtgttgggtcgtttgttcGTCGACCTGCAGCCAAGCTATCGAATTCC, ggggACAACtttgtaTAATAAAGTTGgcggccgcTCTAGAACTAGTGGATCCCC).

Then, the entry vector pENTR-L4-P(tetO)-R1 was established from pProF-PHtetO_2_ by restriction enzyme digestion with PacI (New England Biolabs Japan) and DraI (TaKaRa Bio). The destination vector PB-DEST-R4R3 was established from the PB-TET plasmid (Addgene). PB-TET was digested with SalI (TaKaRa) and XbaI (TaKaRa) to be ligated with a SpeI-XhoI-digested *ccd*B fragment. Then, the two expression vectors were constructed by three-fragment LR cloning using the LR Clonase II enzyme (Thermo Fisher Scientific). PB-P(tetO)-hAscl1-pA-PGK-PuroTK-pA was constructed from four plasmids, as follows: pENTR-L4-P(tetO)-R1, pENTR221-hASCL1 (DNAFORM), pMK-pA-PGKpacTK-pA1, and PB-DEST-R4R3. PB-P(tetO)-hDLX2-pA-floxPGKneo-pA was also constructed from the following four plasmids: pENTR-L4-P(tetO)-R1, pENTR/D-hDLX2 (RIKEN BRC), pMK-pA-floxPGKneopA, and PB-DEST-R4R3.

### Neuronal differentiation by transcription factor overexpression

Based on a previous study (Matsushita et al., 2017), *NEUROG2*-inducible iPSCs were established using the following vectors: PB-TET-PH-lox66FRT-*NEUROG2*, pCMV-HyPBase-PGK-Puro (a gift from Dr. Kosuke Yusa, Wellcome Sanger Institute, Hinxton, U.K.), and PB-CAGrtTA3G-IH. These vectors were cotransfected into iPSCs using Gene Juice Transfection Reagent (Merck). The transfectants were cultured in StemFit AK02N containing 450 μg/ml hygromycin (FUJIFILM Wako Pure Chemical) and 0.1-1.0 μg/ml puromycin (Merck). To establish *ASCL1*- and *DLX2*-inducible iPSCs, we used the following vectors: PB-P(tetO)-hAscl1-pA-PGK-PuroTK-pA, PB-P(tetO)-hDLX2-pA-floxPGKneo-pA, pCMV-HyPBase-PGK-Puro, and PB-CAGrtTA3G-IH. These vectors were cotransfected into iPSCs using Gene Juice Transfection Reagent (Merck). The transfectants were cultured in StemFit AK02N containing 450 μg/ml hygromycin (FUJIFILM Wako Pure Chemical), 0.1-1.0 μg/ml puromycin (Merck), and 100 μg/ml G418 (nacalai tesque).

Feeder-free cultured iPSCs were used for the induction of neuronal differentiation. The cells were dissociated and seeded on culture dishes coated with poly-l-lysine, iMatrix-511 (Laminin-511 E8), and Laminin (R&D Systems). To induce glutamatergic neurons, *NEUROG2*-transduced cells were cultured in induction medium [MHM+B27 containing Y27632, FUJIFILM Wako Pure Chemical; 10 μm DAPT, Merck; 2.5 μm IWP-2; and 2 μg/ml doxycycline (Dox), FUJIFILM Wako Pure Chemical] on day 0. For GABAergic neuron induction, *ASCL1*- and *DLX2*-transfected cells were cultured in induction medium with 80 ng/ml recombinant human sonic hedgehog (R&D Systems). On day 5, the medium was replaced with neuron culture medium [MHM+B27 containing 20 ng/ml brain-derived neurotrophic factor (BDNF), R&D Systems; 10 ng/ml glial cell line-derived neurotrophic factor (GDNF), Alomone Labs; 200 μm l-ascorbic acid, Merck; and 100 μm dibutyryl cyclic adenosine monophosphate, Merck] or function assay medium (BrainPhys Neuronal Medium and N2-A/SM1, STEMCELL Technologies; containing 20 ng/ml BDNF, 20 ng/ml GDNF, 200 μm l-ascorbic acid, and 1 mm dibutyryl cyclic adenosine monophosphate) . Treatment with 2 μm PD0332991 isethionate (Merck) was also performed for both types of neuron induction from day 5 to day 21. The medium was replaced every 4 d after day 5.

### Immunocytochemistry

Cells were fixed with 4% paraformaldehyde for 20 min at room temperature and then incubated with blocking buffer (PBS containing 2% normal fetal bovine serum, 2% normal goat serum, 2% bovine serum albumin, and 0.2% Triton X-100) for 1 h at room temperature. Then, the cells were incubated overnight at 4°C with primary antibodies diluted with blocking buffer without Triton X-100. The cells were washed three times with PBS and then incubated with secondary antibodies with Alexa Fluor 488, Alexa Fluor 555, or Alexa Fluor 647 (1:1000; Thermo Fisher Scientific) and Hoechst 33342 (Merck) for 1–2 h at room temperature. After washing with PBS, the cells were examined with a BZ-9000 microscope (Keyence) and an IN Cell Analyzer 6000 (GE Healthcare). The following antibodies were used as the primary antibodies in this study: Oct4 (1:500; mouse, catalog #sc-5279, Santa Cruz Biotechnology), Nanog (1:500; rabbit, RCAB0004PF, REPLPCELL), SSEA4 (1:500; mouse, catalog #ab16287, Abcam), Tra1-60 (1:500; mouse, MAB4360, Millipore), βIII tubulin (1:500; mouse, T8660, Sigma-Aldrich), α-fetoprotein (AFP; 1:250; mouse, MAB1368, R&D Systems), α-smooth muscle actin (αSMA; 1:300; mouse, A2547, Sigma-Aldrich), microtubule-associated protein 2 (MAP2; 1:1000; rabbit, M4403, Merck), MAP2 (1:1000; mouse, AB5622, Millipore), vesicular glutamate transporter 2 (VGluT2; 1:500; mouse, ab79157, Abcam), GABA (1:500; rabbit, A2052, Sigma-Aldrich), Synapsin I (1:2000; rabbit, S193, Sigma-Aldrich), Homer I (1:500; mouse, 2a8, Synaptic Systems), and Gephyrin (1:500; mouse, 147111, Synaptic Systems).

### High-content image analysis

For morphologic analysis, 96-well plates with V-bottomed conical wells containing 1 EB in each well were imaged on an IN Cell Analyzer 6000 using the 2× objective. EBs were identified based on the contrast with the background; from the EB images obtained, the area and form factor were calculated using IN Cell Developer Toolbox version 1.9 (GE Healthcare).

For fluorescence intensity analysis and neurite length analysis of the EBs, stained plates were imaged on an IN Cell Analyzer 6000 high-content cellular analysis system; only the field containing the EB was selectively collected from each well using the 2× objective, resulting in 1 EB being scored per well. For cell population assays and dendrite length analysis, stained plates were imaged with an IN Cell Analyzer 6000; a set of 5 × 5 fields was collected from each well using the 20× objective. For quantitative analysis of the synaptic puncta, stained plates were imaged with an IN Cell Analyzer 6000; a set of 6 × 6 fields was collected from each well using the 60× objective.

Analysis using IN Cell Developer Toolbox version 1.9 (GE Healthcare) began by identifying intact nuclei stained by the Hoechst dye, which were defined as traced nuclei that were >50 μm^2^ in surface area and with intensity levels that were typical and lower than the threshold brightness of pyknotic cells. Each traced nuclear region was then expanded by 50% and cross-referenced with an endodermal marker (AFP), a mesodermal marker (αSMA), and an ectodermal marker (βIII tubulin) for identification; from these images, the fluorescence intensity of each marker was calculated. Using the expanded nuclear region images, neuron markers (βIII tubulin and MAP2), a glutamatergic neuron marker (VGluT2), and a GABAergic neuron marker (GABA) were identified; from these images, the percentage of each marker was calculated (for neural subtype markers stained as granules, the number of βIII tubulin ^+^ cells containing one or more of the markers was quantified). Using the above-described traced neuronal images of each cell, neurite length, dendrite length, and the number of synaptic puncta (Homer I^+^, Gephyrin^+^, and/or Synapsin I^+^ puncta) were also analyzed.

### Microarray analysis

RNA was extracted with the RNeasy Kit (QIAGEN), and the RNA quality and quantity were assessed using a bioanalyzer (model 2100, Agilent Technologies). Microarray analysis was performed using the Clariom S Assay for humans (Thermo Fisher Scientific). The accession number was referred later. Data were analyzed by Transcriptome Analysis Console 4.0 (Thermo Fisher Scientific). Gene ontology (GO) and pathway analyses were performed using DAVID 6.8 Bioinformatics Resources (https://david.ncifcrf.gov). The data shown in this publication have been deposited in the NCBI Gene Expression Omnibus database (http://www.ncbi.nlm.nih.gov/geo/). The accession number of our microarray analysis data was GSE116820.

### Genetic engineering of *PCDH15*-mutant iPSCs

*PCDH15*-deleted iPSCs were established using the CRISPR/Cas9 system, as previously described (Arioka et al., 2018). In brief, single-guide RNA (sgRNA) expression vectors were constructed using pHL-H1-ccdB-mEF1α-RiH vector (catalog #60601, Addgene) with two oligos containing the sgRNA target site and a universal reverse primer (Table 1). The T7 endonuclease I (T7E1) assay was performed using genomic DNA from HEK293FT cells co-transfected with the sgRNA expression vector and Cas9 expression vector (Addgene ID: 60599) to assess sgRNA activity. The target region of sgRNA was amplified from the genomic DNA using a specific primer set (Table 1). The PCR products were digested using T7E1 (New England Biolabs) after denaturing and reannealing. To establish the isogenic *PCDH15*-deleted iPSC line, sgRNA and Cas9 expression vectors were cotransfected into healthy control iPSCs, NC1032-1-2 (Control 1) and cells were selected with puromycin.

**Table 1.** Used primers for generating isogenic *PCDH15* deletion iPSCs

Targets	Primers	Sequence (5´ → 3´)
sgRNAs construction	sgRNA#1-Fw	GAGACCACTTGGATCCGGACGGCAATCACGAGTGTTGTTTTAGAGCTAGAAATAGCA
	sgRNA#2-Fw	GAGACCACTTGGATCCGTCGCCTCTCATTCAGATTTGTTTTAGAGCTAGAAATAGCA
	sgRNA#3-Fw	GAGACCACTTGGATCCGTGGCAGCTTGATAAGTGAGGTTTTAGAGCTAGAAATAGCA
	sgRNA#4-Fw	GAGACCACTTGGATCCGCGCCTCTCATTCAGATTTTGTTTTAGAGCTAGAAATAGCA
	sgRNA#5-Fw	GAGACCACTTGGATCCGCTCATTCAGATTTTGGGCAGTTTTAGAGCTAGAAATAGCA
sgRNA#universal-Rv		GCCCGGGTTTGAATTCAAAAAAAGCACCGACTCGGTGCCACTTTTTCAAGTTGATAACGGACTAGCCTTATTTTAACTTGCTATTTCTAGCTCTAA
T7E1 assay	Fw	CTCAGTTTACATCCTGACTCAACCAC
	Rv	CCTTCAAACGGCCAAACATAATCTCC

Sequence information of the primers for sgRNAs construction and T7E1 assay. Fw, Forward primer; Rv, reverse primer. Also see Materials and Methods.

**Table 2.** *p* Values of the indicated statistical comparisons

Figures	Measurement	Type of test	comparison	*p* Value
Figure 2*C*	EB size (EB)	Dunnett’s test	Control vs BP1	0.4406
			Control vs SCZ1	0.5982
	EB size (DSi-EB)	Dunnett’s test	Control vs BP1	0.5619
			Control vs SCZ1	0.9784
Figure 2*D*	EB form factor (EB)	Dunnett’s test	Control vs BP1	0.5015
			Control vs SCZ1	0.7476
	EB form factor (DSi-EB)	Dunnett’s test	Control vs BP1	0.0662
			Control vs SCZ1	0.0562
Figure 2*E*	*PCDH15* gene expression	Dunnett’s test	Control vs BP1	0.3861
			Control vs SCZ1	0.3897
	*Reln* gene expression		Control vs BP1	0.7288
			Control vs SCZ1	0.5178
Figure 2*G*	Intensity/area (βIII tubulin)	Dunnett’s test	Control vs BP1	0.9826
			Control vs SCZ1	0.2543
	Intensity/area (αSMA)	Dunnett’s test	Control vs BP1	0.9778
			Control vs SCZ1	0.9238
	Intensity/area (AFP)	Dunnett’s test	Control vs BP1	0.7731
			Control vs SCZ1	0.9345
Figure 2*J*	Neurite length (day 10)	Dunnett’s test	Control vs BP1	0.7746
			Control vs SCZ1	0.8824
	Neurite length (day13)	Dunnett’s test	Control vs BP1	0.0293
			Control vs SCZ1	0.0324
	Neurite length (day16)	Dunnett’s test	Control vs BP1	0.0192
			Control vs SCZ1	0.01
	Neurite length (day22)	Dunnett’s test	Control vs BP1	0.0036
			Control vs SCZ1	0.0025
Figure 3*C*	No. of cells (βIII tubulin^+^/HO^+^)	Tukey’s test	1210B2 vs 201B7	0.9993
			1210B2 vs BP1-1	1
			1210B2 vs BP1-2	1
			1210B2 vs BP2-1	0.0218
			1210B2 vs SCZ1-1	0.5216
			1210B2 vs SCZ1-2	0.875
			201B7 vs BP1-1	0.9975
			201B7 vs BP1-2	0.9989
			201B7 vs BP2-1	0.0036
			201B7 vs SCZ1-1	0.2079
			201B7 vs SCZ1-2	0.5352
			BP1-1 vs BP1-2	1
			BP1-1 vs BP2-1	0.0077
			BP1-1 vs SCZ1-1	0.4016
			BP1-1 vs SCZ1-2	0.8143
			BP1-2 vs BP2-1	0.0042
			BP1-2 vs SCZ1-1	0.3116
			BP1-2 vs SCZ1-2	0.7283
			BP2-1 vs SCZ1-1	0.5238
			BP2-1 vs SCZ1-2	0.1306
			SCZ1-1 vs SCZ1-2	0.9847
	No. of cells (MAP2^+^/βIII tubulin^+^)	Tukey’s test	1210B2 vs 201B7	1
			1210B2 vs BP1-1	0.6317
			1210B2 vs BP1-2	0.9984
			1210B2 vs BP2-1	0.6973
			1210B2 vs SCZ1-1	0.9988
			1210B2 vs SCZ1-2	0.9994
			201B7 vs BP1-1	0.7153
			201B7 vs BP1-2	1
			201B7 vs BP2-1	0.7806
			201B7 vs SCZ1-1	1
			201B7 vs SCZ1-2	1
			BP1-1 vs BP1-2	0.7807
			BP1-1 vs BP2-1	1
			BP1-1 vs SCZ1-1	0.8539
			BP1-1 vs SCZ1-2	0.7703
			BP1-2 vs BP2-1	0.8448
			BP1-2 vs SCZ1-1	1
			BP1-2 vs SCZ1-2	1
			BP2-1 vs SCZ1-1	0.8955
			BP2-1 vs SCZ1-2	0.8325
			SCZ1-1 vs SCZ1-2	1

	No. of cells (VGluT2^+^βIII tubulin^+^/βIII tubulin^+^)	Tukey’s test	1210B2 vs 201B7	0.9629
			1210B2 vs BP1-1	0.2892
			1210B2 vs BP1-2	0.9885
			1210B2 vs BP2-1	0.8416
			1210B2 vs SCZ1-1	0.6553
			1210B2 vs SCZ1-2	0.9756
			201B7 vs BP1-1	0.7818
			201B7 vs BP1-2	0.9999
			201B7 vs BP2-1	0.9996
			201B7 vs SCZ1-1	0.9859
			201B7 vs SCZ1-2	1
			BP1-1 vs BP1-2	0.5003
			BP1-1 vs BP2-1	0.9484
			BP1-1 vs SCZ1-1	0.9956
			BP1-1 vs SCZ1-2	0.6512
			BP1-2 vs BP2-1	0.9885
			BP1-2 vs SCZ1-1	0.9093
			BP1-2 vs SCZ1-2	1
			BP2-1 vs SCZ1-1	0.9998
			BP2-1 vs SCZ1-2	0.9975
			SCZ1-1 vs SCZ1-2	0.9616
Figure 3*D*	*PCDH15* gene expression (iPS)	Dunnett’s test	Control vs BP	0.9527
			Control vs SCZ	0.9354
	*PCDH15* gene expression (neuron)	Dunnett’s test	Control vs BP	0.2862
			Control vs SCZ	0.4102
	*RELN* gene expression (iPS)	Dunnett’s test	Control vs BP	0.1826
			Control vs SCZ	0.3746
	*RELN* gene expression (neuron)	Dunnett’s test	Control vs BP	0.6918
			Control vs SCZ	0.9941
Figure 3*G*	No. of cells (βIII tubulin^+^/HO^+^)	Tukey’s test	1210B2 vs 201B7	0.98
			1210B2 vs BP1-1	0.9914
			1210B2 vs BP1-2	0.9863
			1210B2 vs BP2-1	0.4556
			1210B2 vs SCZ1-1	1
			1210B2 vs SCZ1-2	0.9992
			201B7 vs BP1-1	0.7241
			201B7 vs BP1-2	1
			201B7 vs BP2-1	0.1395
			201B7 vs SCZ1-1	0.9894
			201B7 vs SCZ1-2	0.9993
			BP1-1 vs BP1-2	0.7192
			BP1-1 vs BP2-1	0.7873
			BP1-1 vs SCZ1-1	0.9536
			BP1-1 vs SCZ1-2	0.8677
			BP1-2 vs BP2-1	0.1102
			BP1-2 vs SCZ1-1	0.9937
			BP1-2 vs SCZ1-2	0.9998
			BP2-1 vs SCZ1-1	0.2366
			BP2-1 vs SCZ1-2	0.1646
			SCZ1-1 vs SCZ1-2	0.9999
	No. of cells (MAP2^+^/βIII tubulin^+^)	Tukey’s test	1210B2 vs 201B7	0.9997
			1210B2 vs BP1-1	0.9897
			1210B2 vs BP1-2	0.9829
			1210B2 vs BP2-1	0.5806
			1210B2 vs SCZ1-1	0.9812
			1210B2 vs SCZ1-2	0.9842
			201B7 vs BP1-1	1
			201B7 vs BP1-2	0.9998
			201B7 vs BP2-1	0.8884
			201B7 vs SCZ1-1	0.9037
			201B7 vs SCZ1-2	0.9149
			BP1-1 vs BP1-2	1
			BP1-1 vs BP2-1	0.902
			BP1-1 vs SCZ1-1	0.6069
			BP1-1 vs SCZ1-2	0.6541
			BP1-2 vs BP2-1	0.9712
			BP1-2 vs SCZ1-1	0.6265
			BP1-2 vs SCZ1-2	0.6636
			BP2-1 vs SCZ1-1	0.0941

			BP2-1 vs SCZ1-2	01225
			SCZ1-1 vs SCZ1-2	1
	No. of cells (GABA^+^βIII tubulin^+^/βIII tubulin^+^)	Tukey’s test	1210B2 vs 201B7	0.9415
			1210B2 vs BP1-1	0.2961
			1210B2 vs BP1-2	0.6938
			1210B2 vs BP2-1	0.2707
			1210B2 vs SCZ1-1	0.343
			1210B2 vs SCZ1-2	0.8786
			201B7 vs BP1-1	0.9599
			201B7 vs BP1-2	0.9994
			201B7 vs BP2-1	0.949
			201B7 vs SCZ1-1	0.9747
			201B7 vs SCZ1-2	1
			BP1-1 vs BP1-2	0.9981
			BP1-1 vs BP2-1	1
			BP1-1 vs SCZ1-1	1
			BP1-1 vs SCZ1-2	0.9345
			BP1-2 vs BP2-1	0.9967
			BP1-2 vs SCZ1-1	0.9994
			BP1-2 vs SCZ1-2	0.9993
			BP2-1 vs SCZ1-1	1
			BP2-1 vs SCZ1-2	0.9169
			SCZ1-1 vs SCZ1-2	0.9587
Figure 3*H*	*PCDH15* gene expression (iPS)	Dunnett’s test	Control vs BP	0.2334
			Control vs SCZ1	0.8567
	*PCDH15* gene expression (neuron)	Dunnett’s test	Control vs BP	0.3458
			Control vs SCZ	0.5146
	*RELN* gene expression (iPS)	Dunnett’s test	Control vs BP	0.999
			Control vs SCZ	0.9874
	*RELN* gene expression (neuron)	Dunnett’s test	Control vs BP	0.081
			Control vs SCZ	0.0663
Figure 5*D*	Dendrite length	Dunnett’s test	Control vs BP	0.0001
			Control vs SCZ	0.0002
Figure 5*E*	Homer I puncta No.	Dunnett’s test	Control vs BP	0.0093
			Control vs SCZ	0.009
	Synapsin I puncta No.	Dunnett’s test	Control vs BP	0.0067
			Control vs SCZ	0.0014
	Homer I-Synapsin I puncta No.	Dunnett’s test	Control vs BP	0.0182
			Control vs SCZ	0.0117
Figure 5*H*	Dendrite length	Dunnett’s test	Control vs BP	0.004
			Control vs SCZ	0.0016
Figure 5*I*	Gephyrin puncta No.	Dunnett’s test	Control vs BP	0.0065
			Control vs SCZ	0.0048
	Synapsin I puncta No.	Dunnett’s test	Control vs BP	0.0173
			Control vs SCZ	0.0099
	Gephyrin-Synapsin I puncta No.	Dunnett’s test	Control vs BP	0.0044
			Control vs SCZ	0.0042
Figure 6*E*	Gene expression of *PCDH15*	Student’s *t* test	Control 1 vs PCDH15del (glutamatergic neurons)	0.1757
			Control 1 vs PCDH15del (GABAergic neurons)	0.01
Figure 6*G*	Gene expression of *RELN*	Student’s *t* test	Control 2 vs RELNdel (glutamatergic neurons)	<0.0001
			Control 2 vs RELNdel (GABAergic neurons)	0.0014
Figure 7*B*	No. of cells (βIII tubulin^+^/HO^+^)	Student’s *t* test	Control 1 vs PCDH15del	0.1781
			Control 1 vs RELNdel	0.2969
	No. of cells (MAP2^+^/βIII tubulin^+^)	Student’s *t* test	Control 1 vs PCDH15del	0.0003
			Control 1 vs RELNdel	0.2568
	No. of cells (VGluT2^+^βIII tubulin^+^/βIII tubulin^+^)	Student’s *t* test	Control 1 vs PCDH15del	0.0098
			Control 1 vs RELNdel	0.2754
Figure 7*C*	Dendrite length	Student’s *t* test	Control 1 vs PCDH15del	0.0418
			Control 1 vs RELNdel	0.063
Figure 7*D*	Homer I puncta No.	Student’s *t* test	Control 1 vs PCDH15del	0.0282
			Control 1 vs RELNdel	0.0209
	Synapsin I puncta No.	Student’s *t* test	Control 1 vs PCDH15del	0.2101
			Control 1 vs RELNdel	0.3278
	Homer I-Synapsin I puncta No.	Student’s *t* test	Control 1 vs PCDH15del	0.982
			Control 1 vs RELNdel	0.7786
Figure 7*F*	No. of cells (βIII tubulin^+^/HO^+^)		Control 1 vs PCDH15del	0.1441
			Control 1 vs RELNdel	0.1954
	No. of cells (MAP2^+^/βIII tubulin^+^)		Control 1 vs PCDH15del	0.0281
			Control 1 vs RELNdel	0.4007

	No. of cells (GABA^+^βIII tubulin^+^/βIII tubulin^+^)		Control 1 vs PCDH15del	0.1269
			Control 1 vs RELNdel	0.66
Figure 7*G*	Dendrite length	Student’s *t* test	Control 1 vs PCDH15del	0.0055
			Control 1 vs RELNdel	0.9907
Figure 7*H*	Gephyrin puncta No.	Student’s *t* test	Control 1 vs PCDH15del	0.553
			Control 1 vs RELNdel	0.0452
	Synapsin I puncta No.	Student’s *t* test	Control 1 vs PCDH15del	0.2637
			Control 1 vs RELNdel	0.0431
	Gephyrin-Synapsin I puncta No.	Student’s *t* test	Control 1 vs PCDH15del	0.5136
			Control 1 vs RELNdel	0.3123
Figure 8*B*	Dendrite length (Glutamatergic neurons)	Dunnett’s test	Control vs BP	0.0001
			Control vs SCZ	0.0002
	Homer I puncta No.	Dunnett’s test	Control vs BP	0.0003
			Control vs SCZ	0.001
	Synapsin I puncta No.	Dunnett’s test	Control vs BP	0.0011
			Control vs SCZ	0.0012
	Homer I-Synapsin I puncta No.	Dunnett’s test	Control vs BP	<0.0001
			Control vs SCZ	<0.0001
	Dendrite length (GABAergic neurons)	Dunnett’s test	Control vs BP	0.0021
			Control vs SCZ	0.0027
	Gephyrin puncta No.	Dunnett’s test	Control vs BP	0.0002
			Control vs SCZ	0.0011
	Synapsin I puncta No.	Dunnett’s test	Control vs BP	0.1728
			Control vs SCZ	0.1023
	Gephyrin-Synapsin I puncta No.	Dunnett’s test	Control vs BP	<0.0001
			Control vs SCZ	<0.0001
Figure 8*E*	Spike frequency	Dunnett’s test	Control vs BP (day28)	0.8839
			Control vs SCZ (day28)	0.924
			Control vs BP (day42)	0.7486
			Control vs SCZ (day42)	0.9812
		Paired *t* test	day28 vs day42	0.0104
Figure 8*F*	Spike frequency	Dunnett’s test	Control 1 vs PCDH15del (day28)	0.6129
			Control 2 vs RELNdel (day28)	0.7575
			Control 1 vs PCDH15del (day42)	0.2678
			Control 2 vs RELNdel (day42)	0.9187
		Paired *t* test	day28 vs day42	0.0483
Figure 8*G*	Spike number ratio (CNQX)	Dunnett’s test	Control vs BP	0.0351
			Control vs SCZ	0.0479
	Spike number ratio (AP-5)		Control vs BP	0.1922
			Control vs SCZ	0.1165
	Spike number ratio (GABA)		Control vs BP	0.1204
			Control vs SCZ	0.0071
Figure 8*I*	ΔFmax	Dunnett’s test	1210B2 vs BP1-1	0.9215
			1210B2 vs BP1-2	0.9233
			1210B2 vs BP2-1	1
			1210B2 vs SCZ1-1	0.9999
			1210B2 vs SCZ1-2	0.9997
	Spike frequency	Dunnett’s test	1210B2 vs BP1-1	0.2334
			1210B2 vs BP1-2	0.7435
			1210B2 vs BP2-1	0.9984
			1210B2 vs SCZ1-1	0.9985
			1210B2 vs SCZ1-2	1
Figure 8*J*	Δ*F*max	Student’s *t* test	Control 1 vs PCDH15del	0.7346
			Control 1 vs RELNdel	0.5067
	Spike frequency	Student’s *t* test	Control 1 vs PCDH15del	0.0795
			Control 1 vs RELNdel	0.3771

*p* Values <0.05 were considered to be statistically significant in this study.

### Microelectrode array analysis

The microelectrode array (MEA) was assayed using the Maestro system (Axion Biosystems). Neuronal inductions from iPSCs were performed in 48-well MEA plates coated with poly-l-lysine, iMatrix-511, and laminin. For neuronal differentiation, iPSCs were cultured in induction medium for the first 5 d and then in function assay medium from day 5 until day 28 or day 42. Data were acquired using a sampling rate of 12.5 kHz and filtered using a 200–3000 Hz Butterworth bandpass filter. The detection threshold was set to +6.0 × SD of the baseline electrode noise. Five minutes of activity was subsequently recorded at 37°C. Given the considerable variation in spike count between wells and plates, we focused on active electrodes (electrodes with an average of >5 spikes/min; Isoda et al., 2016), which were certain to make contact with neurons. The number of active electrodes and the mean spike rate per active electrode (spikes per second) was calculated using the spike count file generated by the Axion Integrated Studio program (Axion Biosystems). The spike raster plot was generated by Neural Metric Tool (Axion Biosystems). To determine the responses to the agonist or antagonists of glutamate or the GABA receptor, 50 µm CNQX (Tocris Bioscience), 50 µm AP-5 (Alomone Labs), or 10 µm GABA (nacalai tesque) was applied as treatment, and the activity was recorded.

### Calcium imaging

Neurons were induced from iPSCs by culture in induction medium for 5 d followed by function assay medium from day 5. Calcium imaging analyses were performed on days 28–30. Cells were loaded with 1 μg/ml fluo-8 AM (AAT Bioquest) in recording medium (20 mm HEPES, 115 mm NaCl, 5.4 mm KCl, 0.8 mm MgCl_2_, 1.8 mm CaCl_2_, and 13.8 mm glucose; Dojindo) containing 0.02% Cremophor EL (Dojindo) and incubated for 20 min at 37°C and 5% CO_2_. After washing with PBS, the medium was changed to the recording medium. Changes in fluorescence intensity were measured using an IX83 inverted microscope (Olympus) equipped with an Electron Multiplying CCD Camera (Hamamatsu Photonics) and LED illumination system pE-4000 (CoolLED). We recorded 3500 frames (1 frame: 31–32 ms) per well using the stream acquisition mode. MetaMorph Image Analysis Software (Molecular Devices) was used to analyze the live cell calcium traces. Regions of interest (ROIs) were drawn on cells based on time projection images of the recordings. ROI traces of the time course of changes in fluorescence intensity were generated and used as substrates for subsequent analyses. To adjust for photobleaching, the difference in intensity between the first frame and last frame was calculated and subtracted from the raw intensity. The change in fluorescence intensity over time was normalized as Δ*F*/*F* = (*F* − *F*_0_)/*F*_0_, where *F*_0_ is fluorescence at the starting point of exposure (time 0). Δ*F*max was defined as the difference of the largest change of Δ*F*/*F* associated with each calcium spike.

### Statistical analysis

Statistical analyses were performed using a Dunnett’s or Tukey’s test for multiple comparisons. For comparison between two groups, an unpaired Student’s *t* test or a paired *t* test was used. All statistical analyses were performed using SAS 9.2 (SAS Institute). Probability values (*p* value) <0.05 were considered to be statistically significant. Statistical test results are included in Table 2.

## Results

### Generation of iPSCs from BP patients

**Figure 1.**
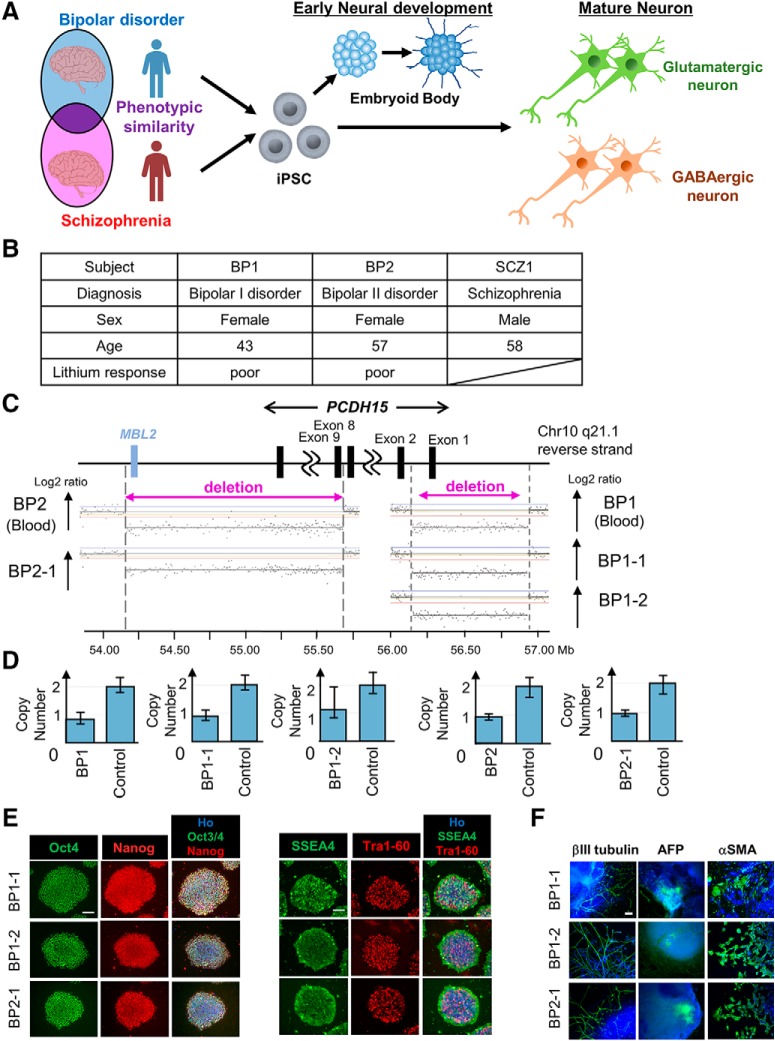
Generation and characterization of iPSCs derived from a BP patient. ***A***, Schematic diagram of the strategy to explore the phenotypes of BP and SCZ *in vitro*. ***B***, Subjects list containing basic information. ***C***, CNVs in chromosome 10 were detected in blood and iPSCs derived from two BP patients by aCGH. ***D***, The exonic deletions of *PCDH15* identified in this study were validated by the TaqMan copy number assays. Bars indicate copy numbers predicted by the TaqMan copy number assays. Capped bars indicated the minimum and maximum copy number calculated for the sample replicate group (*n* = 4). Controls carried no aCGH-detected CNVs of PCDH15 (copy number = 2) and were used to calibrate the assays. ***E***, The generated iPSCs expressed the pluripotent markers Oct4, Nanog, SSEA4, and Tra1-60. Scale bar, 100 μm. ***F***, Representative images of immunocytochemical analysis for *in vitro* three-germ layer differentiation. βIII-Tubulin, αSMA, and AFP are pluripotent markers of the ectoderm, mesoderm, and endoderm, respectively. Blue indicates Ho, and green indicates the pluripotent marker. Scale bar, 100 μm.

BP and SCZ are distinct neuropsychiatric diseases but share a subset of similar pathologies and symptoms (Maggioni et al., 2017). To recapitulate the shared BP and SCZ pathologies *in vitro*, we prepared iPSCs derived from BP and SCZ patients to induce neurons (Fig. 1*A*). We focused on two BP patients (BP1 and BP2), who were evaluated in a high-resolution CNV analysis study (our unpublished observation). The patient characteristics are listed in Figure 1*B* (see also Materials and Methods). Using aCGH, we identified an exonic deletion of *PCDH15* in each patient with BP (Fig. 1*C*). The coordinate of this deletion of BP1 was chr10:56,149,683-56,924,932, and the deletion of BP2 was chr10:54,162,953-55,694,398. The deletion of BP1 included exon 1 of most of the *PCDH15* transcripts, and the deletion of BP2 included *PCDH15* exon 9 to the last exon and *MBL2* (mannose binding lectin 2). These *PCDH15* deletions were validated by the TaqMan copy number assay (Fig. 1*D*). We generated two iPSC clones (BP1-1, BP1-2) from BP1 and one iPSC clone (BP2-1) using episomal plasmids (see Materials and Methods), and we detected the same exonic deletions of *PCDH15* as those detected in blood cells (Fig. 1*C*,*D*). Immunocytochemical analysis demonstrated that our newly established BP-iPSCs expressed the pluripotent markers Oct4, Nanog, SSEA4, and Tra1-60 (Fig. 1*E*). *In vitro* three-germ layer differentiation analysis via EBs also showed the differentiation potentials of the iPSCs into three germ layers (Fig. 1*F*). These results suggest that the BP-iPSCs conserved the patient’s genetic background and had adequate pluripotency.

We also used two previously established iPSC clones (SCZ1-1 and SCZ1-2) derived from an SCZ patient with *RELN* deletion (SCZ1) (Fig. 1*B*; Arioka et al., 2018; Sobue et al., 2018) as SCZ patient-derived iPSCs (SCZ-iPSCs) in subsequent experiments.

### Ectoderm-enriched embryoid body and neuron formation by chemical treatment

There are two major methods for neural differentiation from iPSCs or embryonic stem cells (ESCs). One is the chemical induction method, in which iPSCs are treated with several pharmacological agents or bioactive proteins for differentiation into neurons via EB or neurosphere formation (Han et al., 2011; Espuny-Camacho et al., 2013; Matsumoto et al., 2016). The other is the direct conversion method, involving the overexpression of specific transcription factors (Zhang et al., 2013; Colasante et al., 2015; Sun et al., 2016; Yang et al., 2017). To select the best method for recapitulating the disease-specific mature neuron phenotypes, we used both methods to differentiate iPSCs into neurons (Fig. 1*A*).

**Figure 2.**
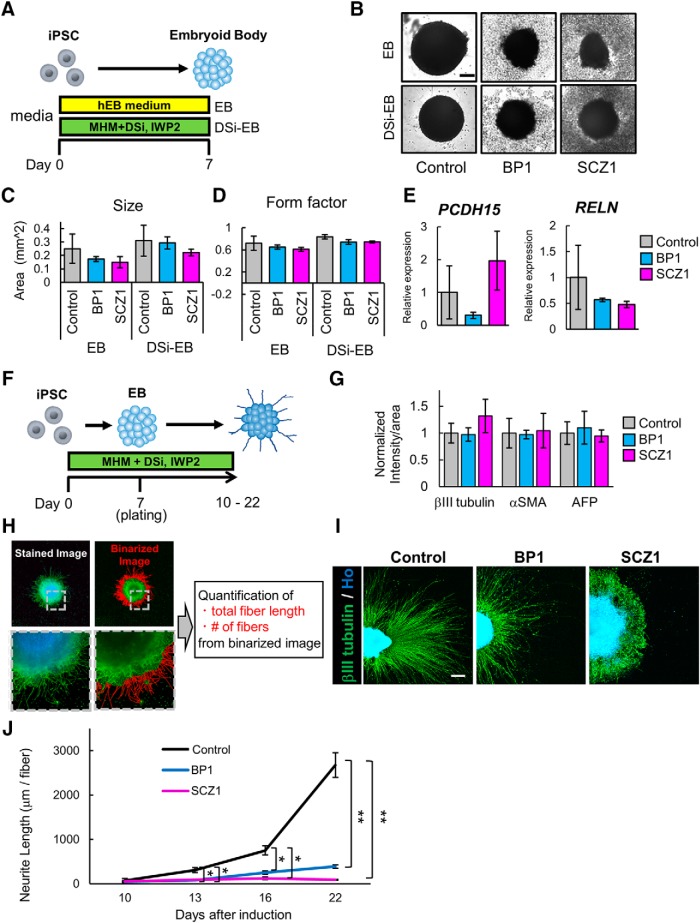
Neuron differentiation via EB formation. ***A***, Overview of the protocol for EB formation. DSi represents SB431542 and LDN193189. ***B***, Representative images of EBs on day 7. Scale bar, 200 μm. ***C***, Quantification of EB sizes (*n* = 3 independent experiments; mean ± SD; Dunnett’s test, no significant differences were observed). ***D***, The form factor of the EBs was calculated as an indicator of roundness. ***E***, Gene expression of iPSCs and DSi-EBs. ***E***, Relative gene expression levels of *PCDH15* and *RELN* in DSi-EBs derived from six control lines, two BP lines (BP1-1 and BP1-2), and two SCZ lines (SCZ1-1 and SCZ1-2; *n* = 3 independent experiments; mean ± SD; Dunnett’s test). Values were normalized to that of the control, which was considered to be 1.0. One sample of 1210B2-derived DSi-EBs, in which *PCDH15* expression was under the detection limit, was removed from the analysis. ***F***, Overview of the protocol for neuron differentiation via EB formation. DSi represents SB431542 and LDN193189. ***G***, Intensity levels of three germ layer markers, namely, βIII-tubulin, αSMA, and AFP. Intensity levels were normalized to that of the control, which was considered to be 1.0 (*n* = 3 independent experiments; mean ± SD; Dunnett’s test among each group, no significant differences were observed). ***H***, Schematic diagram of the analysis protocol. Fiber length and number of βIII tubulin^+^ cells were quantified from binarized image data obtained from stained images. ***I***, Representative images of βIII-tubulin^+^ neurons. Scale bar, 300 μm. ***J***, Time-dependent changes of βIII-tubulin^+^ mean neurite length per neurite fiber (*n* = 3 independent experiments; mean ± SD; *p < 0.05, **p < 0.01; Dunnett’s test among each group). Mean neurite length is shown as the mean length of βIII-tubulin^+^ neurite fiber.

First, we performed chemical treatment. In previous studies, SCZ or BP patient-derived iPSCs exhibited abnormal phenotypes of early-stage neural differentiation when this type of method was used (Madison et al., 2015; Toyoshima et al., 2016). We used BP-iPSCs (BP1-1, BP1-2) and SCZ-iPSCs (SCZ1-1, SCZ1-2) as patient-derived iPSCs. To confirm whether such phenotypes occurred in our iPSC lines, neurons were induced via EB formation. First, we checked the potential of the two types of EB formation. Normal EBs were formed in EB medium (Fig. 2*A*). Another type of EB is induced by dual SMAD inhibition to facilitate neural differentiation (Chambers et al., 2009). DSi-EBs were induced in MHM with two DSis, namely, SB431542 and LDN193189 (Imaizumi et al., 2015; Matsumoto et al., 2016). The Wnt inhibitor IWP-2 was also used for DSi-EB formation to induce anterior regionality of the brain (Fig. 2*A*; Imaizumi et al., 2015; Nehme et al., 2018). Six clones of iPSCs (1210B2, 201B7, eKA3, eTKA4, WD39, and 414C2) were used as healthy control iPSCs. Each group of iPSCs formed EBs by both methods (Fig. 2*B*). To assess EB development, we examined morphometric parameters including size and form factor. The patient-derived iPSCs tended to form smaller and more distorted EBs than the control iPSCs, although there were no significant differences in size and form factor among EBs derived from each group of iPSC lines (Fig. 2*C*,*D*). In addition, the *PCDH15* and *RELN* gene expression levels of DSi-EBs were analyzed by qRT-PCR. The expression of both genes did not change significantly between the control and BP or SCZ, although PCDH15 tended to exhibit low expression in BP samples and RELN exhibited a similar trend in SCZ samples (Fig. 2*E*).

To investigate whether neurons from the patient-derived iPSCs exhibited aberrant phenotypes, we induced neural differentiation via DSi-EB formation. DSi-EBs were plated on culture plates on day 7. Plated DSi-EBs were cultured for up to an additional 15 d in MHM with DSi and IWP-2 (Fig. 2*F*). We used 1210B2 and 201B7 as control iPSCs, and BP-iPSCs (BP1-1, BP1-2) and SCZ-iPSCs (SCZ1-1, SCZ1-2) were used as patient-derived iPSCs. Immunocytochemical analysis showed that the expression levels of markers of the three germ layers, namely, βIII tubulin (ectoderm), αSMA (mesoderm), and AFP (endoderm), in the patient-derived cells did not differ significantly from those in the control cells (Fig. 2*G*). Thus, the capacity of differentiation into the three germ layers among the control and both patient-derived iPSCs was likely to be the same. We then measured the length of neurite extension from the cell clusters. To visualize the neurites, cells were immunostained for βIII tubulin. Binarized images were formed from stained images to quantify the total fiber length of each cell cluster (*a*) and fiber numbers of each cell cluster (*b*) of the βIII tubulin^+^ cells (Fig. 2*H*). Average neurite length was defined as (*a*)/(*b*). The neurite lengths of patient-derived cells were significantly shortened from day 13 onward (Fig. 2*I*,*J*). We found that this method was not suitable for the analysis of mature neurons because of early-stage neuron abnormalities, although these phenotypes may reflect developmental abnormalities of the pathologies of BP and SCZ. Thus, we decided not to perform further analysis using this method.

### Neuronal differentiation of iPSCs by overexpression of transcription factors

**Figure 3.**
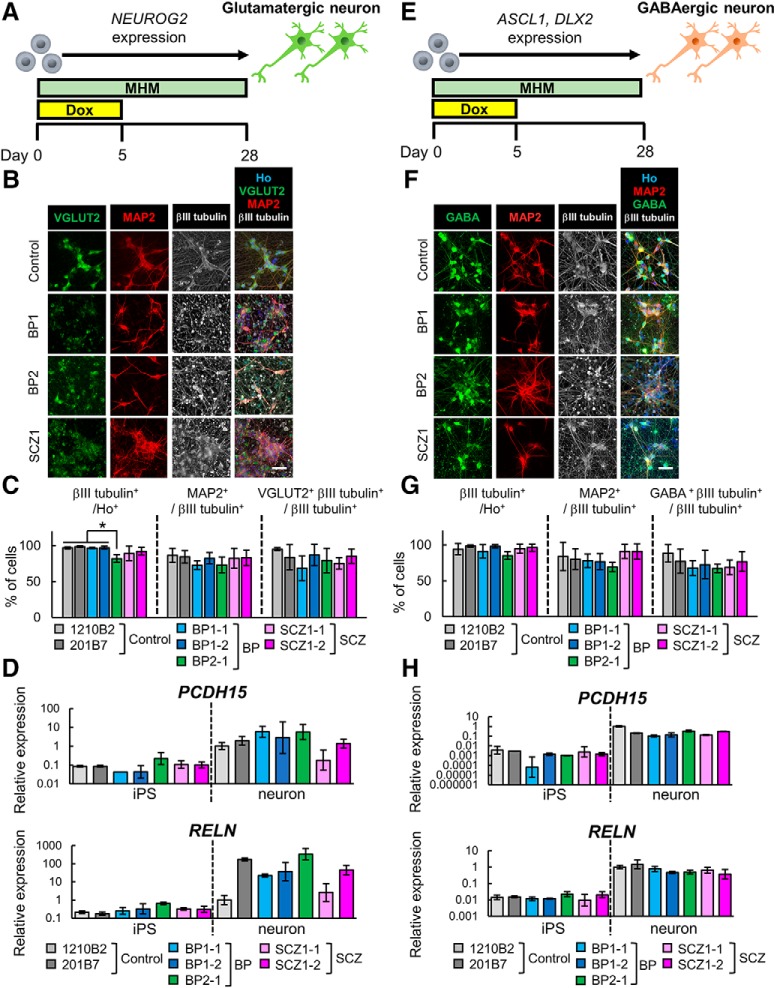
Neuron differentiation by transcription factor overexpression**. *A***, Overview of the protocol for differentiation into glutamatergic neurons. ***B***, Immunocytochemical analysis of neuronal markers (VGluT2, MAP2, and βIII tubulin). Scale bar, 40 μm. ***C***, Ratio of positive cells for each marker; βIII tubulin^+^ cells/all cells (βIII tubulin^+^/Ho^+^), MAP2^+^ cells/βIII tubulin^+^ cells (MAP2^+^/ βIII-tubulin^+^), and VGluT2 and βIII tubulin double^+^ cells/βIII tubulin^+^ cells (VGLUT2^+^βIII tubulin^+^/βIII tubulin^+^). (*n* = 3-6 independent experiments; mean ± SD; *p < 0.05; Tukey’s test, the population of βIII tubulin^+^ cells in BP2-1-derived neurons was significantly lower than those in 1210B2-, 201B7-, BP1-1-, and BP1-2-derived neurons). ***D***, Relative gene expression levels of *PCDH15* (primer set1) and *RELN* in *NEUROG2*-transduced iPSCs and induced neurons (*n* = 3 independent experiments; mean ± SD; Dunnett’s test among iPSCs and among neurons, no significant differences were observed). Values were normalized to that for 1210B2-derived neurons, which was considered to be 1.0. Some iPSC samples, in which *PCDH15* expression was under the detection limit, were removed from the analysis. Specifically, two samples of BP1-1, and one sample of 1210B2, 201B7, BP2-1, and SCZ1-1 were excluded from the analysis. ***E***, Overview of the protocol for differentiation into GABAergic neurons. ***F***, Immunocytochemical analysis of neuronal markers (MAP2, GABA, and βIII tubulin). Scale bar, 40 μm. ***G***, Ratio of positive cells for each marker; βIII tubulin^+^ cells/all cells (βIII tubulin^+^/Ho^+^), MAP2^+^ cells/βIII tubulin^+^ cells (MAP2^+^/βIII tubulin^+^), and GABA and βIII-tubulin double-positive cells/βIII tubulin^+^ cells (GABA^+^βIII tubulin^+^/βIII tubulin^+^). (*n* = 3-6 independent experiments; mean ± SD; Tukey’s test; no significant differences were observed). ***H***, Relative gene expression levels of *PCDH15* and *RELN* in *ASCL1*- and *DLX2*-transduced iPSCs and induced neurons. (*n* = 3 independent experiments; mean ± SD; Dunnett’s test among iPSCs and among neurons; no significant differences were observed). Values were normalized to that for 1210B2-derived neurons, which was considered to be 1.0. Some iPSC samples, in which *PCDH15* expression was under the detection limit, were removed from the analysis. Specifically, each one sample of 1210B2 and BP2-1 was excluded from the analysis.

We next tested the direct method for neuronal conversion of iPSCs by transcription factor overexpression. This method can induce specific neuron subtypes. Previous studies have shown that *Neurog2* overexpression induced glutamatergic neuron differentiation (Zhang et al., 2013), and co-overexpression of *ASCL1* and *DLX2* induced GABAergic neuron differentiation (Yang et al., 2017). Although these studies used lentivirus as a tool for introducing transcription factors, use of the PiggyBac transposon vector system enabled the establishment of transgenic cells that can be easily stored and used for experiments. One report using the PiggyBac system for the introduction of transcription factors into human ESCs resulted in highly efficient neural differentiation (Matsushita et al., 2017). Therefore, we established transgenic iPSC lines in which transcription factors can be induced by Dox. We used 1210B2 and 201B7 as healthy control iPSCs, while we used BP-iPSCs (BP1-1, BP1-2, and BP2-1) and SCZ-iPSCs (SCZ1-1, SCZ1-2) as patient-derived iPSCs. *NEUROG2* expression vector-transduced iPSCs were established for glutamatergic neuronal induction, and *Ascl1*- and *DLX2*-transduced iPSCs were established for GABAergic neuronal induction (see Materials and Methods). The iPSCs were treated with Dox for 5 d and cultured in MHM for 28 d (Fig. 3*A*,*E*). Immunocytochemical analysis showed that the neuronal marker βIII tubulin was expressed in most of the cells induced from control iPSCs (control neurons), BP-iPSCs (BP neurons), and SCZ-iPSCs (SCZ neurons; Fig. 3*B*,*C*,*F*,*G*). The conversion efficiency of iPSCs into βIII tubulin^+^ neurons was close to 100% in all lines, except line BP2-1 which still showed >80% efficiency (Fig. 3*C*). βIII tubulin^+^ neurons derived from *NEUROG2*-transduced iPSCs expressed the mature neuronal marker MAP2 and the glutamatergic neuronal marker VGLUT2 at similar ratios in each line (Fig. 3*B*,*C*). Next, the *PCDH15* and *RELN* gene expression levels of *NEUROG2*-transduced cells (iPSCs and induced glutamatergic neurons) were analyzed by qRT-PCR. We used the primer sets targeting the deletion of *PCDH15* in BP1 or *RELN* in SCZ1. Both *PCDH15* and *RELN* were substantially upregulated on neuronal induction but were not differentially expressed between control, BP, SCZ iPSCs, or neurons (Fig. 3*D*). Induced neurons derived from *ASCL1*- and *DLX2*-transduced iPSCs expressed MAP2 and the GABAergic neuronal marker GABA at similar ratios in each line (Fig. 3*F*,*G*). The *PCDH15* and *RELN* gene expression levels of *ASCL1*- and *DLX2*-cotransduced cells (iPSCs and induced GABAergic neurons) are shown in Figure 3*H*. The expression of both genes was upregulated in GABAergic neurons compared with undifferentiated iPSCs. Neither *PCDH15* nor *RELN* expression changed significantly in patient-derived cells. These results showed that this method enabled both healthy and patient-derived iPSCs to efficiently differentiate into subtype-specific and mature neurons. Thus, we chose this method for further analyses.

### Characterization of gene expression in neurons induced from iPSCs

**Figure 4.**
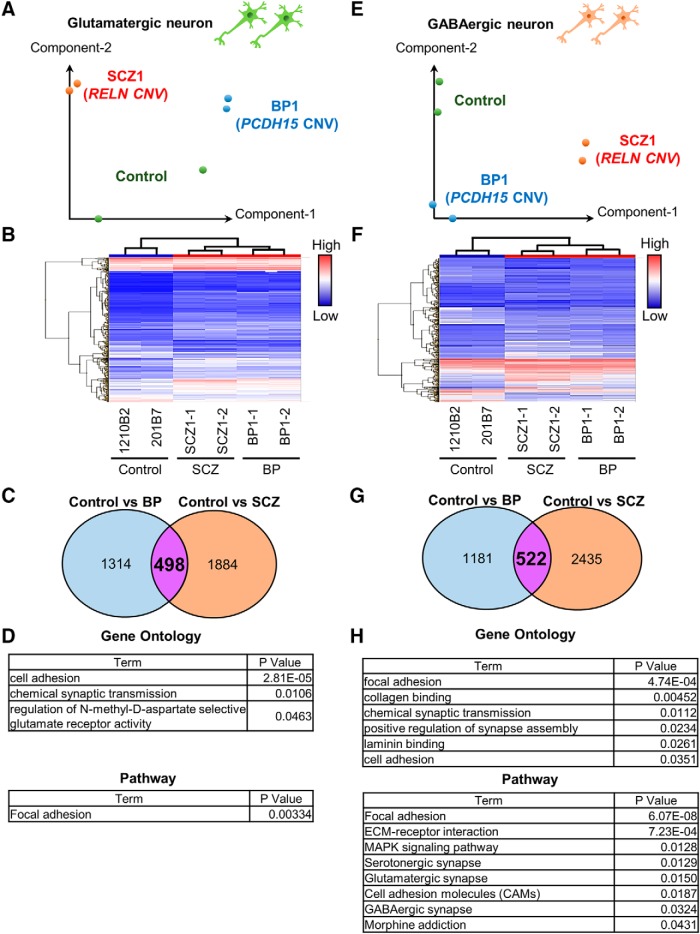
Comparison of the global gene expression profiles. ***A***, PCA of the gene expression data of glutamatergic neurons. ***B***, Hierarchical clustering analysis of DEGs in glutamatergic neurons. ***C***, Venn diagram of DEGs of BP or SCZ glutamatergic neurons compared with control neurons (fold change, >2.0). A total of 498 genes were common between the two groups compared. ***D***, Featured GO and pathway terms for the 498 common DEGs among the control vs BP and control vs SCZ. Adhesion- and neuron-associated terms were extracted. ***E***, PCA plot of the gene expression data of GABAergic neurons. Green, control (1210B2, 201B7); blue, BP; red, SCZ. ***F***, Hierarchical clustering analysis of DEGs in GABAergic neurons. ***G***, Venn diagram of DEGs of BP or SCZ GABAergic neurons in comparison with control neurons (fold change, >2.0). A total of 522 genes were common among the two comparisons. ***H***, Featured GO and pathway terms for 522 common DEGs among the control vs BP and control vs SCZ. Adhesion- and neuron-associated terms were extracted.

To identify the characteristics of neurons induced from patient-derived iPSCs, the global gene expression profiles of neurons induced from control-iPSCs (1210B2 and 201B7) and patient-derived iPSCs (BP1-1, BP1-2, SCZ1-1, and SCZ1-2) were examined by a microarray analysis. Principal component analysis (PCA; Fig. 4*A*,*E*) and hierarchical analysis (Fig. 4*B*,*F*) showed that the control, BP, and SCZ neurons were placed in different groups. In particular, the profiles of the BP and SCZ neurons were closer to each other than to that of the control neurons (Fig. 4*B*,*F*). We next identified the differentially expressed genes (DEGs) in neurons induced from patient-derived iPSCs compared with control neurons (fold change, >2.0). We identified 1812 genes of BP glutamatergic neurons and 2382 genes of SCZ glutamatergic neurons that were upregulated or downregulated compared with the control neurons (Fig. 4*C*). For the GABAergic neurons, the expression levels of 1703 genes of BP neurons and 2357 genes of SCZ neurons were changed (Fig. 4*G*). To explore the cellular functions associated with the DEGs in the BP and SCZ neurons, GO analysis and pathway analysis were performed. Featured GO terms and pathways of common DEGs between BP and SCZ are shown in Figure 4, *D* and *H*. In both glutamatergic and GABAergic neurons, multiple GO terms and pathways related to cell adhesion and neural function were enriched compared with control neurons (Fig. 4*D*,*H*).

### Neurons induced from patient-derived iPSCs exhibited shorter dendrites and reduced formation of excitatory and inhibitory synapses

**Figure 5.**
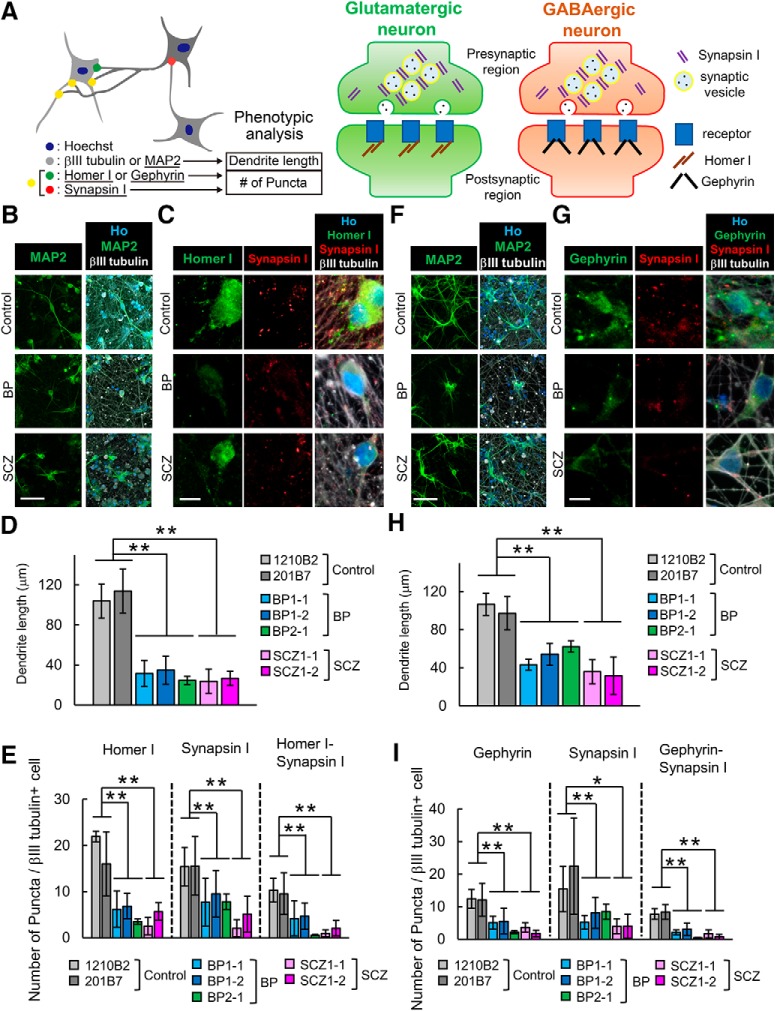
Neurons induced from patient-derived iPSCs exhibit abnormal phenotypes of dendrite length and synapse formation. ***A***, Schematic diagram of the protocol for phenotypic analysis of dendrite lengths and number of synaptic markers of the neurons. ***B***, ***C***, Representative images of immunocytochemical analysis of a dendrite marker (MAP2; scale bar, 60 μm; ***B***) and synaptic markers (Homer I, Synapsin I; scale bar, 10 μm; ***C***) in glutamatergic neurons. ***D***, Quantitative analysis of dendrite length in glutamatergic neurons (*n* = 3-6 independent experiments; mean ± SD; **p < 0.01; Dunnett’s test). ***E***, Quantitative analysis of the number of synaptic marker puncta in glutamatergic neurons (*n* = 3-6 independent experiments; mean ± SD; *p < 0.05, **p < 0.01; Dunnett’s test). Homer I puncta, Synapsin I puncta and Homer-Synapsin I colocalized puncta on βIII-tubulin^+^ cells were counted. ***F***, ***G***, Representative images of immunocytochemical analysis of a dendrite marker (MAP2; scale bar, 60 μm; ***F***) and synaptic markers (Gephyrin, Synapsin I; scale bar, 10 μm; ***G***) in GABAergic neurons. ***H***, Quantitative analysis of dendrite length in GABAergic neurons (*n* = 3-6 independent experiments; mean ± SD; **p < 0.01, Dunnett’s test). ***I***, Quantitative analysis of the number of synaptic marker puncta in glutamatergic neurons (*n* = 3-6 independent experiments; mean ± SD; *p < 0.05, **p < 0.01, Dunnett’s test). Gephyrin puncta, Synapsin I puncta, and Gephyrin-Synapsin I colocalized puncta on βIII tubulin^+^ cells were counted.

Microarray and GO analyses suggested that genes involved in cell adhesion may contribute to pathogenetic mechanisms (Fig. 4). Cell adhesion is a key event for neurite extension and synapse formation (Kiryushko et al., 2004; Missler et al., 2012). In addition, previous studies using postmortem human brains showed reduced spine density and dendrite length in the brains of BP and SCZ patients (Glantz and Lewis, 2000; Konopaske et al., 2014). To investigate whether BP and SCZ neurons have similar phenotypes, we determined the dendrite lengths and synaptic densities of induced neurons by immunocytochemical analysis with an IN Cell Analyzer 6000. Dendrite length was measured as the length of the region immunostained with the dendrite marker MAP2 (Fig. 5*A*). Then, to detect the number of morphological synapses in glutamatergic neurons, we counted the puncta of Synapsin I and Homer I as presynaptic or postsynaptic markers. For GABAergic neurons, we determined the number of puncta positive for Synapsin I and Gephyrin, which is a postsynaptic scaffolding protein found in GABAergic synapses (Fig. 5*A*). MAP2^+^ dendrites of BP and SCZ neurons were significantly shorter than those of control neurons in both glutamatergic and GABAergic neurons (Fig. 5*B*,*D*,*F*,*H*). In addition, fewer puncta of Homer I and Synapsin I on glutamatergic neurons were detected in BP and SCZ neurons than in control neurons (Fig. 5*C*,*E*). The number of puncta of GABAergic Synapsin I and Gephyrin were also reduced in BP and SCZ neurons compared with control neurons (Fig. 5*G*,*I*). Moreover, the number of colocalized puncta between presynaptic and postsynaptic markers was also significantly decreased in BP and SCZ neurons (Fig. 5*E*,*I*). Thus, the number of synapses decreased in both BP and SCZ neurons. These results suggest that neurons induced from patient-derived iPSCs exhibit synapse- and dendrite-related phenotypes that are consistent with the phenotypes often observed in patients with psychiatric disorders and can thus serve as disease models of psychiatric disorders *in vitro*.

### Establishment of isogenic PCDH15-deleted iPSCs

**Figure 6.**
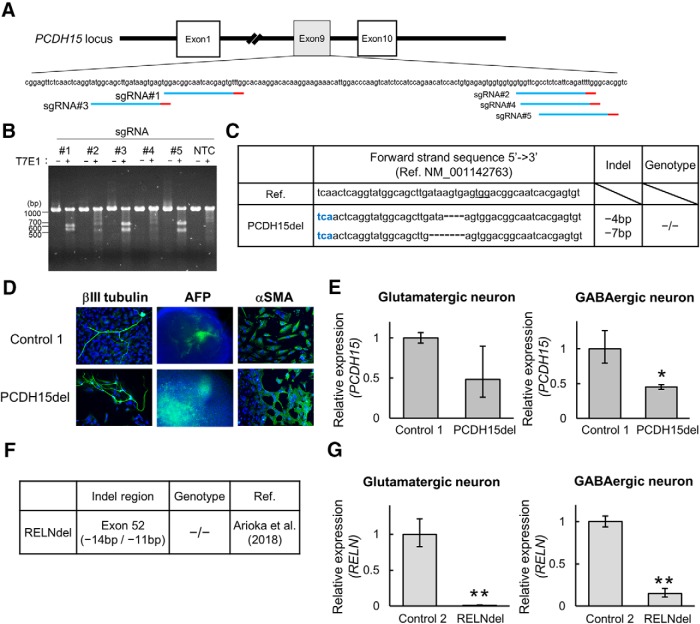
Generation of isogenic *PCDH15* deletion iPSCs by targeted genome editing. ***A***, The target sites of CRISPR-sgRNAs in exon 9 of the *PCDH15* gene (NM_001142763). Red bars represent PAM (protospacer adjacent motif) sequences. ***B***, Analysis of CRISPR-sgRNA activity by the T7E1 assay using HEK293FT. NTC, No transfection. Among the constructed sgRNAs, sgRNA#3 showed the strongest cleavage activity. ***C***, Indel pattern of the isogenic line using CRISPR-sgRNA#3. Blue letters represent stop codon. ***D***, Representative images of immunocytochemical analysis for *in vitro* three-germ layer differentiation. Blue indicates Ho and green indicates the pluripotent marker (βIII-tubulin, αSMA, and AFP). Scale bar, 100 μm. ***E***, Relative gene expression levels of *PCDH15* (primer set2) in glutamatergic or GABAergic neurons on day 28 (*n* = 3 independent experiments; mean ± SD; *p < 0.05, Student’s *t* test). ***F***, Information of isogenic *RELN*-deleted iPSCs generated in the previous study (Arioka et al., 2018). ***G***, Relative gene expression levels of *RELN* in induced glutamatergic or GABAergic neurons on day 28 (*n* = 3 independent experiments; mean ± SD; **p < 0.01, Student’s *t* test).

It is difficult to perform large-scale analyses because of the infrequency of patients carrying particular CNVs. Moreover, line-to-line variability caused by different genetic backgrounds complicate genotype–phenotype correlation. Thus, we sought to establish mutant and control iPSCs by targeted genome editing using the CRISPR/Cas9 system. To generate *PCDH15*-deleted iPSCs, sgRNAs were constructed in exon 9 (Fig. 6*A*). According to the results of the T7E1 assay (see Materials and Methods), sgRNA#3 showed the strongest cleavage activity among the five sgRNA candidates (Fig. 6*B*). We obtained an isogenic line with the homozygous *PCDH15* deletion (PCDH15del) from healthy control NC1032-1-2 (Control 1; Fig. 6*C*). PCDH15del mutant iPSCs showed the capacity to differentiate into three germ layers as well as Control 1 (Fig. 6*D*). Then, we analyzed the expression level of *PCDH15* by qRT-PCR using the primer set targeting the deleted region. The mRNA level of *PCDH15* was decreased by about 50% in PCDH15del-induced neurons (PCDH15del neurons) when normalized to Control 1-induced neurons (Fig. 6*E*). We also prepared an isogenic line with the homozygous *RELN* deletion (RELNdel) that was established from 201B7 (Control 2) in a previous study (Arioka et al., 2018; Fig. 6*F*). Remarkably, the expression level of *RELN* in RELNdel-induced neurons (RELNdel neurons) was much lower than that in Control 2-induced neurons (Fig. 6*G*).

### Dendritic and synaptic characterization of isogenic PCDH15- or RELN-deleted neurons

**Figure 7.**
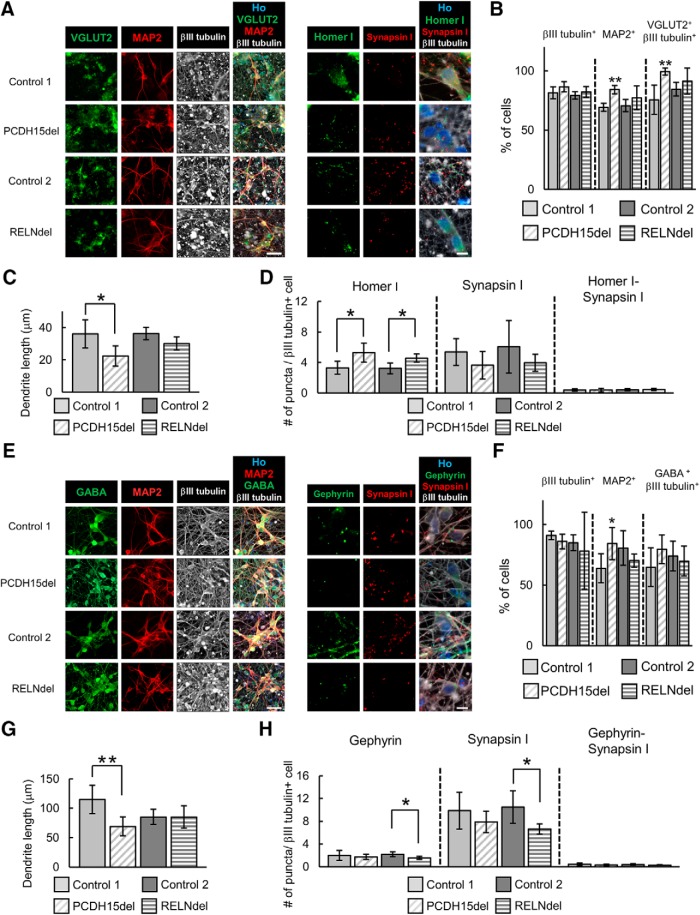
Isogenic *PCDH15* or *RELN* deleted neurons showed partial phenotypes of dendrite and synapse formation. ***A***, Representative images of immunocytochemical analysis of neuronal markers (scale bar, 40 μm) and synaptic markers (scale bar, 10 μm) in glutamatergic neurons. ***B***, Ratio of positive cells for each marker; βIII tubulin^+^ cells/all cells (βIII tubulin^+^), MAP2^+^ cells/βIII tubulin^+^ cells (MAP2^+^), and VGLUT2 and βIII tubulin double-positive cells/βIII tubulin^+^ cells (VGluT2^+^βIII tubulin^+^). (*n* = 4–6 independent experiments; mean ± SD; **p < 0.01, Student’s *t* test between each pair of control and isogenic lines). ***C***, Quantitative analysis of dendrite length in glutamatergic neurons (*n* = 4–6 independent experiments; mean ± SD; *p < 0.05, Student’s *t* test). ***D***, Quantitative analysis of the number of synaptic marker puncta in glutamatergic neurons (*n* = 3–6 independent experiments; mean ± SD; *p < 0.05, Student’s *t* test). ***E***, Representative images of immunocytochemical analysis of neuronal markers (scale bar, 40 μm) and synaptic markers (scale bar, 10 μm) in GABAergic neurons. ***F***, Ratio of positive cells for each marker; βIII tubulin^+^, MAP2^+^, and GABA and βIII tubulin double-positive cells/βIII tubulin^+^ neuronal cells (GABA^+^βIII tubulin^+^). (*n* = 4–6 independent experiments; mean ± SD; *p < 0.05, Student’s *t* test between Control 1 and PCDH15del or between Control 2 and RELNdel). ***G***, Quantitative analysis of dendrite length in GABAergic neurons (*n* = 4–6 independent experiments; mean ± SD; **p < 0.01, Student’s *t* test). ***H***, Quantitative analysis of the number of synaptic marker puncta in GABAergic neurons (*n* = 4–6 independent experiments; mean ± SD; *p < 0.05, Student’s *t* test).

To investigate whether our isogenic gene-edited iPSC-derived neurons recapitulate patient iPSC-derived neurons, we performed phenotypic analyses focusing on their dendrite length and synapse formation using the same method as performed in Figure 5. Both glutamatergic and GABAergic neurons were efficiently induced from PCDH15del and RELNdel (Fig. 7*A*,*B*,*E*,*F*). While some differentiation characteristics were confirmed, >60% of cell populations constantly differentiated into our target neurons in all cell lines we tested (Fig. 7*B*,*F*).

Regarding glutamatergic neurons, MAP2^+^ dendrites of PCDH15del neurons were significantly shorter than those of Control 1 neurons, while RELNdel neurons tended to form shorter dendrites than Control 2 neurons, although the difference was not statistically significant (*p* = 0.063; Fig. 7*C*). Unlike patient-derived neurons, the number of Homer I puncta was increased in both PCDH15del neurons and RELNdel neurons compared with Control 1 or 2 neurons (Fig. 7*D*). The number of Synapsin I puncta and colocalized puncta of Homer I and Synapsin I tended to be lower in isogenic lines, although the changes were not significant (Fig. 7*D*). Regarding GABAergic neurons, MAP2^+^ dendrite shortening was observed only in PCDH15del neurons (Fig. 7*G*), while both Gephyrin and Synapsin I puncta were significantly decreased only in RELNdel neurons (Fig. 7*H*). The number of colocalized puncta was not significantly different in either isogenic line (Fig. 7*H*). Based on these findings, isogenic gene-edited lines partially recapitulated the structural phenotypes observed in patient-derived lines.

### Assessment of neuronal function of patient-derived and isogenic iPSCs-derived neurons

**Figure 8.**
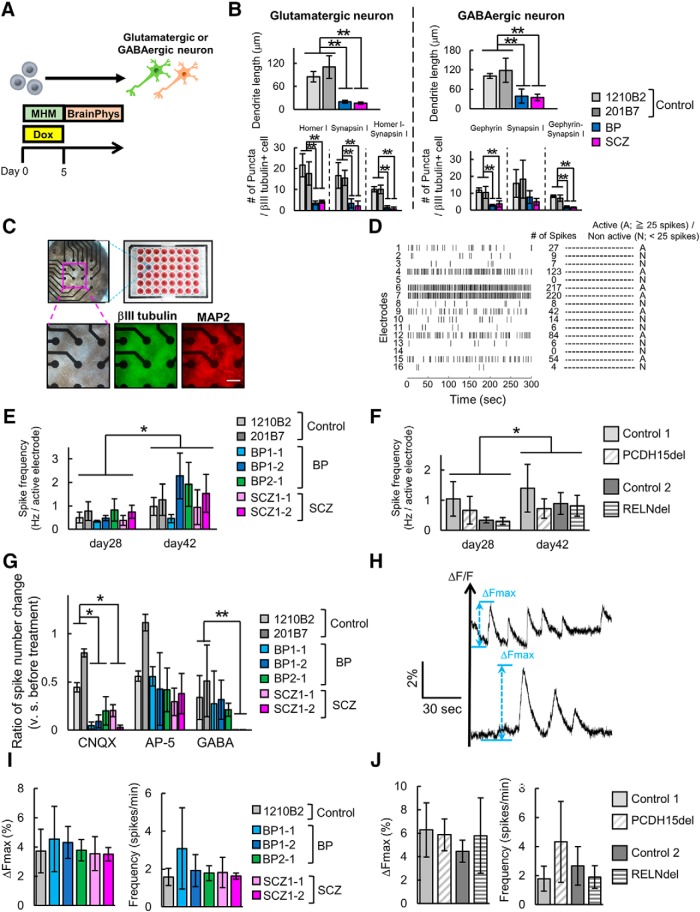
Spontaneous activity of neurons induced from patient-derived or isogenic iPSCs. ***A***, Overview of the protocol for neuronal differentiation for functional analysis. ***B***, Quantitative analysis of dendrite length and synaptic markers puncta in neurons cultured in BrainPhys for differentiation on day 28 (*n* = 3–4 independent experiments; mean ± SD; **p < 0.01, Dunnett’s test among control, BP, and SCZ neurons; BP: BP1-2, SCZ: SCZ1-2). ***C***, Overview of MEA plate and representative images of neurons induced from iPSCs on the 48-well MEA plates. Bright-field image and immunocytochemical images of neuron markers. Scale bar, 200 μm. ***D***, Representative image of raster plot and definition of active electrodes. ***E***, Spike frequency of control or patient-derived glutamatergic neurons on day 28 and day 42 (*n* = 4–6 independent experiments; mean ± SD; Dunnett’s test among Control, BP, and SCZ neurons, no significant differences were observed; paired *t* test between day 28 and day 42, *p < 0.05). ***F***, Spike frequency of isogenic iPSC-derived glutamatergic neurons (*n* = 3–4 independent experiments; mean ± SD; Dunnett’s test among Control, BP, and SCZ neurons, no significant differences were observed; paired *t* test between day 28 and day 42, *p < 0.05). ***G***, Relative change in the total number of spikes after drug treatment in glutamatergic neurons on day 42 (*n* = 3 independent experiments; mean ± SD; *p < 0.05, **p < 0.01, Dunnett’s test). ***H***, Representative image of calcium spikes and display of parameters (Δ*F*max and calcium spike numbers). ***I***, Δ*F*max and calcium spike frequency in control or patient-derived GABAergic neurons (*n* = 3–6 independent experiments; mean ± SD; Dunnett’s test among each line, no significant differences were observed). ***J***, Δ*F*max and calcium spike frequency in control or patient-derived GABAergic neurons (*n* = 4–6 independent experiments; mean ± SD; Student’s *t* test, no significant differences were observed).

We showed that patient-derived neurons or isogenic gene-edited neurons displayed structural phenotypes. However, it remains unknown whether these neurons show differences in functional activity. Thus, we investigated their neuronal function by assaying spontaneous neuronal activity. To clearly assess neuronal activity, we evaluated the effect of BrainPhys medium, which was developed to support neurophysiological activity (Bardy et al., 2015) on neuronal phenotypes we detected (Fig. 8*A*). Even by culturing with BrainPhys, BP and SCZ neurons showed the recapitulated phenotypes of dendrite shortening and decreased synapse number and for both glutamatergic and GABAergic neurons (Fig. 8*B*). Based on these results, we used BrainPhys as the neuron culture media for functional analysis.

For MEA analysis, we induced neurons from iPSCs directly on the MEA plates at a high density (Fig. 8*C*). Spontaneous activity was detected by 16 electrodes per well in the MEA plate, and the activities of neurons were recorded at each electrode (Fig. 8*D*). In glutamatergic neurons, active electrodes were stably detected on day 28 after induction, while few active electrodes appeared in GABAergic neurons on day 28 (data not shown). In spike frequency of glutamatergic neurons on day 28 and day 42, there were no significant differences in spike frequency among control, BP, and SCZ groups, although the time-dependent increase in spike frequencies of each group (Fig. 8*E*). Isogenic *PCDH15*-deleted or *RELN*-deleted neurons also showed no significant differences compared with Control 1 or Control 2 both on day 28 and day 42, except for the time-dependent increase in spike frequencies (Fig. 8*F*). These results indicate that spontaneous neuronal activity was maintained at a comparable level among glutamatergic neurons whether they were control neurons, patient-derived neurons, or isogenic neurons.

Next, we investigated receptor reactivity by treatment with a glutamate receptor antagonist (AMPA receptor antagonist CNQX and NMDA receptor antagonist AP-5) or GABA receptor agonist (GABA). By using induced glutamatergic neurons, we measured changes in the total number of spikes per well before and after treatment. We found that the spike number was significantly decreased both in BP and SCZ neurons after CNQX treatment, but not after AP-5 treatment compared with Control neurons (Fig. 8*G*). Similarly, GABA treatment resulted in a significant decrease in spike number in SCZ neurons compared with Control neurons (Fig. 8*G*). These results suggest that BP and SCZ neurons have higher sensitivities in AMPA receptor and GABA receptor stimulation, which might lead to the maintenance of spontaneous activity of neurons.

In GABAergic neurons, 6 weeks of differentiation enabled neurons to have active spikes in most patient-derived and isogenic lines. However, some cell lines did not show sufficient neuronal activity to be analyzed (data not shown). Then, we used a calcium imaging system to detect the GABAergic neuron activity with higher sensitivity. We used 1210B2 as a healthy control for comparison with patient-derived lines. We recorded spontaneous calcium spikes from GABAergic neurons on day 28 and focused on the following parameters: Δ*F*max and spike number (Fig. 8*H*; see also Materials and Methods). There were no significant differences both in Δ*F*max and spike frequency among control- and patient-derived lines (Fig. 8*I*). Isogenic neurons also showed no significant differences in these parameters compared with controls (Fig. 8*J*). These results of calcium imaging thus suggest that the spontaneous activity of GABAergic neurons is comparable among control, patient-derived, and isogenic neurons, indicating some compensatory mechanism for structural abnormalities.

## Discussion

It is challenging to elucidate the molecular mechanisms and general pathologies of BP and SCZ for the following two reasons: the genetic background complexity of these disorders and the difficulty of pathological recapitulation. However, neurons induced from patient-derived iPSCs can overcome these problems. In particular, the use of iPSCs derived from patients with disease-associated CNVs is a promising strategy (Flaherty et al., 2017; Rutkowski et al., 2017). In the present study, we focused on the following two CNVs: the heterozygous deletion of *PCDH15* and of *RELN*. We used newly generated iPSCs derived from BP patients with *PCDH15* deletion and differentiated the BP-iPSCs (*PCDH15*-deleted) and SCZ-iPSCs (*RELN*-deleted) into neurons efficiently. We identified several neuronal abnormalities in neurons induced from patient-derived iPSCs.

In this study, we used the following two types of methods for neural differentiation: induction by chemical treatment or overexpression of transcription factors. Neurons induced from patient-derived iPSCs using the chemical treatment method exhibited abnormal phenotypes of neurite extension in the early differentiation stage. Although these phenotypes may support the neurodevelopmental hypothesis for BP and SCZ (O'Shea and McInnis, 2016; Owen and O'Donovan, 2017), this differentiation method is not suited for the analysis of adult and specific pathologies for medical treatment. On the other hand, we showed that transcription factor overexpression is suitable for the preparation of specific types of mature neurons and analysis of the phenotypes of clinical pathologies. *NEUROG2* overexpression induced glutamatergic neurons, and *ASCL1* and *DLX2* co-overexpression induced GABAergic neurons, from iPSCs efficiently, which enabled analysis of the phenotype of each type of neuron. In addition, this type of method is expected to be applicable for electrophysiological analysis of specific neurons or functional analysis of neural networks by coculturing different types of neurons (Sun et al., 2016). However, the induction of specific subtypes of GABAergic neurons is also needed for further understanding of the pathologies. It was reported that *ASCL1* and *DLX2* overexpression induced various subtypes of GABAergic neurons, such as calretinin^+^, somatostatin^+^, and parvalbumin^+^ neurons (Yang et al., 2017). Although parvalbumin^+^ neurons have important roles in BP and SCZ (Wang et al., 2011; Marin, 2012), the existing method can induce parvalbumin^+^ neurons at only low levels (Sun et al., 2016; Wen, 2017; Yang et al., 2017). Therefore, an efficient method to induce parvalbumin^+^ neurons is required for further investigation of the roles of GABAergic neurons in BP and SCZ.

Interestingly, we identified similar phenotypes of decreased MAP2^+^ dendrite length and synapse number between BP and SCZ neurons and between glutamatergic and GABAergic neurons. These phenotypes seem to be associated with the common and general pathologies in BP and SCZ in terms of neurite extension and synapse formation, similar to the phenotypes observed in the brains of patients. Our microarray analysis suggested that these common phenotypes were associated with cell adhesion. PCDH15 participates in cell–cell adhesion of hair cells at the inner ear by forming tip-link filaments (Jaiganesh et al., 2018), while there are no reports on the precise role of PCDH15 in neural adhesion. Reelin is also involved in cell adhesion. Reelin-dependent signaling pathways regulate neural adhesion (Sekine et al., 2012; Hirota and Nakajima, 2017). Thus, PCDH15 and Reelin are expected to play key roles in neural adhesion to regulate dendrite or synapse formation. Indeed, it is well known that cell adhesion plays important roles in synapse formation and neurite extension (Missler et al., 2012). Previous studies have suggested that cell adhesion molecules are associated with BP and SCZ (O'Dushlaine et al., 2011; Sakurai, 2017). In addition, if such a basic system of neural development is associated with these phenotypes, the phenotypes may be associated with a broader range of psychiatric disorders. Although, based on these observations, cell adhesion is expected to play important roles in the pathologies of BP and SCZ, it remains unknown whether these phenotypes reflect common pathologies in general patients or are specific to certain genetic backgrounds because of two limitations. First, the number of donors was limited in this study, and further studies are warranted to confirm our findings. Second, the *PCDH15* or *RELN* mRNA levels did not decrease significantly in neurons induced from patient-derived iPSCs according to our qRT-PCR data in contrast to isogenic homozygous *PCDH15*-deleted or *RELN*-deleted neurons. A previous report using *NEUROG2* overexpression-induced neurons from iPSCs of SCZ patients with heterozygous gene deletion obtained a similar result (Flaherty et al., 2017). Therefore, heterozygous gene deletion does not always indicate the downregulation of gene expression in neurons. Considering that these genes tended to be downregulated in patient-derived DSi-EBs, although these changes were not significant, it is possible that gene expression in patient-derived cells was different from that in the control cells during differentiation.

For further insights into the relationship between CNVs and phenotypes, we established and analyzed isogenic *PCDH15*-deleted or *RELN*-deleted iPSCs using genome-editing technology. Although the isogenic lines showed dendritic or synaptic phenotypes partially correlated with those of patient iPSC-derived neurons, these phenotypes of isogenic neurons were weaker. These results suggest that CNVs as well as other factors contribute to these phenotypes. We also confirmed that other clinically important CNVs were not present in the patients, although it is still possible that an unknown genetic background affected the phenotypes or clinical pathologies because BP and SCZ are highly polygenic disorders. If large-scale genome-editing technologies that allow for the rescue of CNVs become available in the future, the contributions of CNVs can be clearly and directly proved. Under these conditions, patient-derived iPSCs may be a better tool than isogenic iPSCs for modeling BP and SCZ.

In addition, we performed functional analysis by measuring spontaneous neural activity to investigate whether the observed structural phenotypes led to functional abnormalities. However, we did not identify any functional differences in spontaneous activity in patient or isogenic neurons. Although our neurons seemed to be immature compared with *in vivo* neurons, which might make it difficult to detect functional differences, our data suggest that the electrophysiological functions of patient-derived glutamatergic neurons were compensated by the enhancement of sensitivity in their receptors. Although structural abnormalities do not always cause functional defects because a compensatory mechanism might contribute to functional maintenance, detailed electrophysiological analyses are warranted in future studies. We also showed that our neurons were electrically active to some extent. Therefore, our *in vitro* model is suitable for further electrophysiological analyses to elucidate the functional significance of CNVs. Also, coculture of both types of our neurons (glutamatergic and GABAergic) or coculture with glial cells, which is expected to promote further functional maturation and neural network formation, may support this approach in the future studies.

For various diseases, potential therapeutic drugs were identified from phenotypic screening using induced tissues from patient-derived iPSCs (Okano and Yamanaka, 2014; Wainger et al., 2014; Hino et al., 2017; Hosoya et al., 2017; Imamura et al., 2017; Kondo et al., 2017; Fujimori et al., 2018; Tabata et al., 2018). Thus, phenotype-recapitulated *in vitro* model using iPSC technology is available for exploring new therapeutic agents. Our model showing disorder-related phenotypes can be a useful tool for identifying novel drug candidates.

Overall, our approach for the induction of specific neurons from patient-derived iPSCs with disease-related genetic polymorphisms enabled us to analyze pathology-associated phenotypes. Our *in vitro* model, which may show general phenotypes of psychiatric disorders, is expected to be applicable for further elucidation of molecular mechanisms or drug discovery.

## Synthesis

Reviewing Editor: Julie Andersen, Buck Institute Decisions are customarily a result of the Reviewing Editor and the peer reviewers coming together and discussing their recommendations until a consensus is reached. When revisions are invited, a fact-based synthesis statement explaining their decision and outlining what is needed to prepare a revision will be listed below.

This revised manuscript studying the possible effect CNV's in the genes RELN and PCDH15 have in psychiatric patients deserves applaud. The authors sincerely addressed the reviewer comments to enhance their study, and created a more complete story by adding advanced and technical experiments. We believe after making the following minor revisions (none being experiments), the manuscript will be ready for publication.

1. In line 14, the authors describe their observations as “general phenotypes for BP and SCZ”, and maintain that perspective throughout their study. Instead, we suggest framing their observations as “general phenotypes of psychiatric disorders” as both RELN and PCDH15 are not known to be specific to either disease.

2. Starting at line 88, the authors describe the 2nd BP patient iPSC line. In the description, they detail that the subject had hypomanic episodes (but no mention of full manic episodes) as well as other symptomology that may indicate the subject may have a lesser version of bipolar disorder, such as bipolar 2 disorder (even though she was diagnosed as BP1). We suggest the authors include a remark addressing this possible incongruency.

3. After line 660, “We did not find any functional differences in patient or isogenic neurons”, can the authors please add a possible explanation of their results being due to their neurons being immature compared to their in vivo counterparts. And that as differentiation protocols become better at generating more mature neural cultures, these cell lines may display functional differences

4. In figure 1D, the authors should list if there are statistical tests for each histogram and if there is significance.

5. For Figure 2B-J, the authors must explain why they only performed these tests with one iPSC line for both BP and SCZ, as it negates the value of the having the additional lines if they are not being utilized. This figure would be much more impactful if its findings were consistent across all the psychiatric lines, and specifically with the isogenic lines. We will not ask the author's to perform these additional experiments, but the authors must address this choice in the manuscript.

6. Figure 2 I&J do not appear consistent with one another. The plot of neurite length implies the psychiatric lines generate neurites that are over 10 times shorter than healthy controls, but the images in panel I do not show that. We ask the authors either re-examine the validity of their neurite length calculations or provide an explanation for this discrepancy.

7. Figure 4A-B, can the authors label the genes in the PCA diagrams?

8. Figure 6E-G. The histograms' y-axis should be labeled with their respective genes of interest. Also in E, looking at histograms and error bars in the GLUT/PCDH15, it appears there should be statistical significance, especially compared to some of the other graphs (ie Fig. 7E).

9. The statistical significance found in Figure 7C,D,F,G,H are hard to believe based of their mean values and error bars. We ask the authors review their statistical analyses to verify the appropriate tests were performed.

10. Adding to point 3, the MEA data in figure 8D seems to indicate that the cultures had yet to reach an in vivo level of maturity based off their limited spiking activity, and could explain why no functional differences were found.

11. Figure 8G, can the authors clarify the y-axis title, is the “spike number change” in Hz, percentage, frequency, etc...?

12. THIS POINT IS OF HIGHEST IMPORTANCE IN OUR REVIEW. In table 2, the authors list all of their statistical tests. Many of the tests appear to be inappropriate for the experiment performed, or there are unexplained variations in the chosen tests between similar experiments.

a. Specifically, in multiple comparison tests, the authors oscillate between Dunnett and Tukey tests. We ask the authors be consistent between the analyses to maintain an ad hoc versus post hoc appearance.

b. For Figures 7F-H, 8B, & 8J it is inappropriate for the authors to use T-tests, and instead should use a test that considers either multiple samples and/or comparisons.

c. The authors oscillate between SD and SEM error bars, we ask the authors pick a consistent error bar to not confuse the future readers of this study.

## Author Response

Point-by-Point Responses to editor and reviewers' comments

We appreciate the editor and reviewers for evaluating our manuscript (#eN-NWR-0403-18) entitled “In vitro modeling of the bipolar disorder and schizophrenia using patient-derived induced pluripotent stem cells with copy number variations of PCDH15 and RELN.” We found the insightful comments made by the reviewers to be very helpful, and we have addressed the issues raised.

We hope that you will find revised manuscript suitable for publication in eNeuro.

Response to the comments by Reviewers:

(Comment 1) In line 14, the authors describe their observations as “general phenotypes for BP and SCZ”, and maintain that perspective throughout their study. Instead, we suggest framing their observations as “general phenotypes of psychiatric disorders” as both RELN and PCDH15 are not known to be specific to either disease.

(Response) We appreciate the reviewer's insightful suggestion. Although we mainly focused on BP and SCZ in this study using iPSCs derived from the patients with these disorders, RELN and PCDH15 can be associated with other disorders as the reviewer pointed out. In fact, both RELN and PCDH15 were also said to be related to ASD. Thus, according to the comment, we added the sentences that the observed phenotypes could be general in psychiatric disorders.

(Comment 2) Starting at line 88, the authors describe the 2nd BP patient iPSC line. In the description, they detail that the subject had hypomanic episodes (but no mention of full manic episodes) as well as other symptomology that may indicate the subject may have a lesser version of bipolar disorder, such as bipolar 2 disorder (even though she was diagnosed as BP1). We suggest the authors include a remark addressing this possible incongruency.

(Response) Thank you for your kind suggestion. According to your comment, we have considerably checked her previous clinical record, and we could not find her severe manic episodes which are necessary for the diagnosis of Bipolar I disorder. Thus, we have revised the diagnosis from Bipolar I disorder to Bipolar II disorder. We corrected the patient information in the manuscript and in Figure 1B.

(Comment 3) After line 660, “We did not find any functional differences in patient or isogenic neurons”, can the authors please add a possible explanation of their results being due to their neurons being immature compared to their in vivo counterparts. And that as differentiation protocols become better at generating more mature neural cultures, these cell lines may display functional differences.

(Comment 10) Adding to point 3, the MEA data in figure 8D seems to indicate that the cultures had yet to reach an in vivo level of maturity based off their limited spiking activity, and could explain why no functional differences were found.

(Response to above two comments) We appreciate the reviewer's important comments. As the reviewer pointed out, our in vitro neurons seemed to be functionally immature compared to in vivo neurons, and more matured neurons may show functional differences. We think co-culture of both glutamatergic neurons and GABAergic neurons or co-culture with glial cells may support further functional maturation or neural network formation. On the other hand, some compensatory mechanisms might also contribute to functional maintenance in patient neurons as suggested in Figure 8G. These issues should be considered in our future studies. We discussed this point and added explanation.

(Comment 4) In figure 1D, the authors should list if there are statistical tests for each histogram and if there is significance.

(Response) Thank you for the thoughtful comment. We analyzed the data using CopyCaller Software (Applied Biosystems). In this software, at least 7 subjects of the same copy number (in this case, PCDH15- or RELN-deleted patients) were required to calculate confidence value, which show an estimate of the confidence that the assigned copy number was the true copy number. Since the subject number was limited, we did not perform the statistical analysis. However, the purpose of this assay is to confirm the CNVs which we identified using array CGH (Figure 1C). And the results of taqman copy number assays (Figure 1D) clearly supported the results of array CGH. Therefore, we believe that the validity of CNVs were assessed in Figure 1C, D.

(Comment 5) For Figure 2B-J, the authors must explain why they only performed these tests with one iPSC line for both BP and SCZ, as it negates the value of the having the additional lines if they are not being utilized. This figure would be much more impactful if its findings were consistent across all the psychiatric lines, and specifically with the isogenic lines. We will not ask the author's to perform these additional experiments, but the authors must address this choice in the manuscript.

(Response) We appreciate the thoughtful and kind comment. Basically, our purpose in Figure 2 was to determine whether the method of neuron induction via EB formation by chemical treatment was suitable for analyzing mature neuron phenotypes. We picked out one donor with BP or SCZ from which two iPSC clones were established, which allowed for considering the variation between clones. In other words, we used two iPSC clones for each psychiatric group.

As a result, the observed phenotypes suggested that this method was not suitable for analysis of mature neurons even though the phenotypes reflected neurodevelopmental pathologies of the disorders. Thus, we did not perform further analysis using isogenic lines. We added the sentences that mentioned this point in the manuscript.

(Comment 6) Figure 2 I&J do not appear consistent with one another. The plot of neurite length implies the psychiatric lines generate neurites that are over 10 times shorter than healthy controls, but the images in panel I do not show that. We ask the authors either re-examine the validity of their neurite length calculations or provide an explanation for this discrepancy.

(Response) We appreciate for the instructive comment. We detected neurites and quantified the neurite length from stained images as shown in Figure 2H. We reconfirmed that the calculations were correctly performed. In Figure 2I, the neurites of the psychiatric lines, especially BP1, might look longer in comparison with their measured values. However, their neurites included many short neurites, and resulted in showing the low averaged values. In addition, we excluded NPC-like migrated cells and cell-crowded regions expressing both βIII-tubulin and Ho for high-accuracy analysis of neurites (Figure 2H). Therefore, we succeeded in accurately visualizing/quantifying their pathologies using our neurite-specific analysis system.

(Comment 7) Figure 4A-B, can the authors label the genes in the PCA diagrams?

(Response) According to the comment, we added the genetic characteristics (PCDH15 CNV or RELN CNV) in the PCA diagrams in Figure 4A, E to clarify the samples information.

(Comment 8) Figure 6E-G. The histograms' y-axis should be labeled with their respective genes of interest. Also in E, looking at histograms and error bars in the GLUT/PCDH15, it appears there should be statistical significance, especially compared to some of the other graphs (ie Fig. 7E).

(Response) Thank you for the kind suggestion. We added the labels of gene names according to the comment. Regarding Figure 6E, we showed the error bars representing SEM (and SD error bars were used in Figure 7). Due to this, it might look there was significant difference compared to Figure 7E. We unified error bars in SD error bars in all figures (please also refer the response to Comment 12C).

(Comment 9) The statistical significance found in Figure 7C,D,F,G,H are hard to believe based of their mean values and error bars. We ask the authors review their statistical analyses to verify the appropriate tests were performed.

(Response) We apologize for any confusion. We used SD error bars, which could overlap with each other if there is significant difference between two groups, while SEM error bars cannot overlap each other when there is significant difference. We used both SD and SEM error bars in the manuscript, which might confuse the reviewers. We reconfirmed that the results of statistical analysis were correct. Then, we unified error bars in SD error bars in all figures (please also refer the response to comment 12C).

(Comment 11) Figure 8G, can the authors clarify the y-axis title, is the “spike number change” in Hz, percentage, frequency, etc...?

(Response) Thank you for the comment. The graph in Figure 8G showed relative spike number changes before and after the treatment. We altered the y-axis title as follows: “Ratio of spike number change (v. s. before treatment)” to clarify it.

(Comment 12) THIS POINT IS OF HIGHEST IMPORTANCE IN OUR REVIEW. In table 2, the authors list all of their statistical tests. Many of the tests appear to be inappropriate for the experiment performed, or there are unexplained variations in the chosen tests between similar experiments.

a. Specifically, in multiple comparison tests, the authors oscillate between Dunnett and Tukey tests. We ask the authors be consistent between the analyses to maintain an ad hoc versus post hoc appearance.

b. For Figures 7F-H, 8B, & 8J it is inappropriate for the authors to use T-tests, and instead should use a test that considers either multiple samples and/or comparisons.

c. The authors oscillate between SD and SEM error bars, we ask the authors pick a consistent error bar to not confuse the future readers of this study.

(Response) We appreciate the reviewer's important comments. We addressed the concerns as follows.

(a) As the reviewer pointed out, it is better to use consistent tests as much as possible. In this study, we used Dunnett's or Tukey's test for multiple comparisons depending on the purpose. Dunnett's test was mainly utilized when we wanted to assess whether or not there were significant changes in each patient group compared with the control group in most figures. On the other hand, we used Tukey's test when we evaluated the ratios of marker-positive cells (Figure 3C, 3G) to compare each clone with each other in these figures because we wanted to assess not the differences from control but the variation in induction efficiency among each clone. Regarding Figure 2G, although we originally used Tukey's test, Dunnett's test seems to be more suitable. Thus, we reanalyzed using Dunnett's test in Figure 2G.

(b) As the reviewer pointed out, we performed Dunnett's test in Figure 8B. Regarding Figure 7F-H and 8J, we made comparisons between Control 1 and PCDH15del or between Control 2 and RELNdel. Because these two pairs (Control 1-PCDH15del and Control 2-RELNdel) or two comparisons were independent of each other, we think that T-tests are appropriate for each comparison.

(c) As the reviewer suggested, using both types of error bar may confuse the future readers. We unified error bars in SD error bars in all figures (SEM error bars were originally used in Figure 2E, 3D, 3H, 6E, 6G).
